# A Descriptor for Non‐Arrhenius Ion Transport Enables Design of Sulfide Superionic Conductors

**DOI:** 10.1002/anie.4940262

**Published:** 2026-09-19

**Authors:** Han Zhou, Xiaoxuan Chen, Hermann Tempel, Yong Yang, Boryann Liaw, Shicheng Yu, Rüdiger‐A. Eichel

**Affiliations:** ^1^ Institute of Energy Technologies – Fundamental Electrochemistry (IET‐1) Forschungszentrum Jülich Jülich Germany; ^2^ Institute of Physical Chemistry RWTH Aachen University Aachen Germany; ^3^ College of Energy Xiamen University Xiamen China; ^4^ High Power Research Laboratory Sparks Nevada USA; ^5^ Faculty of Mechanical Engineering RWTH Aachen University Aachen Germany

**Keywords:** activation energy dispersion, Meyer–Neldel deviation, non‐Arrhenius ion transport, physics‐informed learning, sulfide solid electrolytes

## Abstract

Non‐Arrhenius ion transport is increasingly observed in solid electrolytes but remains difficult to compare across materials or use in screening. A curated database of 867 conductivity‐temperature entries from thio‐LISICON and LGPS‐type sulfides is used to introduce Meyer–Neldel deviation (MND), a physics‐informed, material‐level descriptor of temperature‐dependent changes in apparent activation energy. More than 40% of the analyzed sulfide electrolytes show notable deviations from linear Arrhenius behavior. MND separates low‐conductivity/high‐barrier and superionic/low‐barrier regimes associated with ionic‐radius mismatch, electronegativity difference, configurational entropy, and mixing thermodynamics, while providing a more informative learning target than single‐value activation energies. Sensitivity analyses indicate that material‐level trends remain reasonably robust to variations in conductivity, reference temperature, and sampling density. Application to a reprocessed dataset of 160 compositions and 2,410 conductivity‐temperature entries across seven electrolyte families supports broader applicability. MND‐guided screening of 171 Li_10_Ge*_x_* Si*_y_* Sn*_z_* P_2_S_12_ compositions prioritizes Li_10_Ge_0.3_Si_0.15_Sn_0.55_P_2_S_12_, which exhibits a room‐temperature conductivity of 7.21 mS cm^−^^1^, an apparent activation energy of 0.288 eV, and weak non‐Arrhenius behavior consistent with prediction. Evaluation of a second composition further supports the multistage screening strategy. These results position MND as a chemically interpretable representation for quantifying, learning, and screening temperature‐dependent ion transport.

## Graphical abstract

A curated database of sulfide electrolytes reveals widespread non‐Arrhenius ion transport. Meyer–Neldel deviation (MND) quantifies this behavior, distinguishes low‐conductivity and superionic regimes, and guides composition screening. An experimentally validated candidate exhibits room‐temperature conductivity of 7.21 mS cm^−1^, an apparent activation energy of 0.288 eV, and weak non‐Arrhenius behavior.

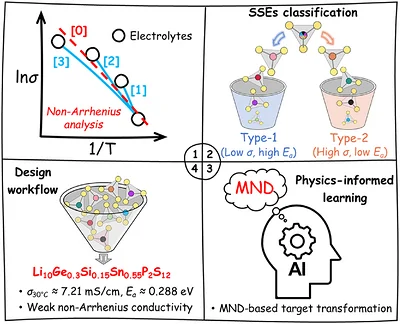

## 1 Introduction

Ionic conductivity is a critical metric for evaluating solid electrolytes because it directly influences the power capability and operating stability of all‐solid‐state batteries [1, 2, 3, 4]. Although liquid electrolytes such as LiPF_6_ in carbonate solvents readily achieve conductivities near 10 mS cm^−1^ at room temperature [5], most crystalline or glassy solid‐state electrolytes remain well below this benchmark. Sulfide electrolytes have recently narrowed this gap, exemplified by Li_10_GeP_2_S_12_ (LGPS), which delivers room‐temperature ionic conductivity approaching 12 mS cm^−1^ and has stimulated extensive exploration of superionic conductors [6, 7, 8]. Yet, high room‐temperature conductivity alone is not sufficient for electrolyte design if ion transport evolves non‐trivially with temperature.

Temperature‐dependent electrochemical impedance spectroscopy (EIS) is the standard tool for probing ionic transport [9, 10], and is typically interpreted using the Arrhenius relation

(1) $$\begin{array}{c} \sigma =\frac{\sigma_{0}}{T}\;e^{-\frac{E_{a}}{k_{B}T}} \end{array}$$

where σ represents the ionic conductivity at a given temperature *T*. σ_0_ is the pre‐exponential factor, often assumed to be a constant for a specific material. *k*_B_ is the Boltzmann constant. *E*_a_ is interpreted as the migration barrier. Ideal Arrhenius behavior corresponds to a linear ln(σ*T*) versus 1/*T* relationship, implying a single apparent migration barrier [11, 12]. However, a growing body of measurements over extended temperature ranges shows that many promising sulfide conductors do not obey such a simple description. Instead, the apparent activation energy can vary with temperature, giving rise to pronounced Arrhenius curvature [13, 14, 15, 16, 17, 18]. Early evidence was reported for AgI‐Ag_2_S‐B_2_S_3_‐SiS_2_ glasses [14], and similar behavior was later observed in glass‐ceramic LGPS [19], where partial amorphization altered the apparent barrier across the measured temperature range, resulting in non‐Arrhenius behavior. Even highly crystalline systems show analogous effects. Li_10_SnP_2_S_12_ exhibits distinct grain and grain‐boundary transport barriers from −140°C to 80°C [20], while solid‐solution Li_10+_ *_δ_* Ge_1+_ *_δ_* P_2‐_ *_δ_* S_12_ (*δ*  = 0.05 and 0.35) displays a progressive change in Arrhenius slope from −80°C to 400°C, with *E*_a_ decreasing from 26 to 7 kJ mol^−1^ [21]. Together, these observations indicate that non‐Arrhenius ion transport is not an isolated anomaly, but a recurring feature across glassy, glass‐ceramic, and crystalline sulfide electrolytes when sufficiently broad temperature windows are examined [22].

Despite increasing experimental evidence, non‐Arrhenius ion transport is still rarely treated as a design‐relevant property in solid electrolytes. Consequently, activation energies extracted from narrow temperature windows are often compared as though they were intrinsic material constants, even though they may depend strongly on the fitting range and transport regime being sampled. Likewise, extrapolating a near‐ambient Arrhenius fit to broader operating conditions can lead to substantial conductivity misestimation, with implications for impedance budgeting, thermal management, fast‐charging behavior, and durability prediction. What is currently missing is a quantitative framework that can capture non‐Arrhenius transport in a form that is comparable across materials, chemically interpretable, and usable for predictive materials screening.

To address this need, an extensive database of sulfide solid electrolytes (SSEs) was assembled, comprising 867 conductivity‐temperature entries and 25 physicochemical descriptors [8, 20, 21, 23, 24, 25, 26, 27, 28, 29, 30, 31, 32, 33, 34, 35, 36, 37, 38, 39, 40, 41, 42, 43, 44, 45, 46, 47, 48, 49]. More than 40% of the entries deviate notably from linear Arrhenius behavior away from ambient conditions, indicating that non‐Arrhenius ion transport is a widespread yet under‐quantified feature of sulfide electrolytes. Inspired by the Meyer–Neldel rule [50, 51, 52], a quantitative descriptor, namely the Meyer–Neldel deviation (MND), is introduced here to capture within‐material variations in apparent activation energy and thereby condense nonlinear transport behavior into a single interpretable quantity. Unlike conventional Meyer–Neldel analysis, which correlates activation energies and pre‐exponential factors across related materials, MND uses the same (*E*_a_, lnσ_0_) parameter space to quantify the dispersion generated by temperature‐pair fits within an individual material. The resulting MND distribution reveals two distinct sulfide transport regimes, Type‐1 SSEs with comparatively low conductivity and high activation barriers, and Type‐2 SSEs with high conductivity and low activation barriers. These regimes correlate with ionic‐radius mismatch, Pauling electronegativity difference, configurational entropy, and mixing thermodynamics, linking temperature‐dependent transport behavior to chemically meaningful descriptors. When used as a physics‐informed target, MND also improves supervised learning by retaining temperature‐dependent information that is lost in conventional single‐value activation‐energy labels and thereby enables more effective recovery of structure‐transport relationships from materials descriptors.

The robustness of MND was evaluated against conductivity uncertainty, reference‐point selection, and temperature‐sampling density, and its transferability was examined using a reprocessed literature dataset spanning seven solid‐electrolyte families. Within the original sulfide domain, descriptor and MND‐based criteria were applied to 171 Li_10_Ge*_x_* Si*_y_* Sn*_z_* P_2_S_12_ compositions. Li_10_Ge_0.3_Si_0.15_Sn_0.55_P_2_S_12_ was prioritized among the descriptor‐eligible candidates and experimentally shown to exhibit a room‐temperature ionic conductivity of 7.21 mS cm^−1^ (cold‐pressed), an apparent activation energy of approximately 0.288 eV, and weak non‐Arrhenius behavior in close agreement with prediction. A second experimentally examined composition provides an additional test of the multistage screening strategy.

## 2 Results and Discussion

### 2.1 Sulfide Solid Electrolyte Database

Figure 1a defines the chemical scope of this study, which is intentionally restricted to thio‐LISICON (*Pnma*) and LGPS‐type SSEs (*P4_2_/nmc*) [53]. These two families share sulfide frameworks composed of tetrahedrally coordinated XS_4_ units (X = P, Ge, Si, Sn, Al, Sb, Ga, and Zn) but span a broad range of compositions and transport performances, making them a suitable domain for evaluating non‐Arrhenius transport within a chemically related space. Restricting the analysis to these sulfide frameworks reduces confounding from fundamentally different anion chemistries and diffusion topologies, while still preserving substantial variation in conductivity, activation barrier, and site chemistry. Halide‐substituted systems are therefore excluded from the present analysis [54, 55, 56, 57].

**FIGURE 1:**
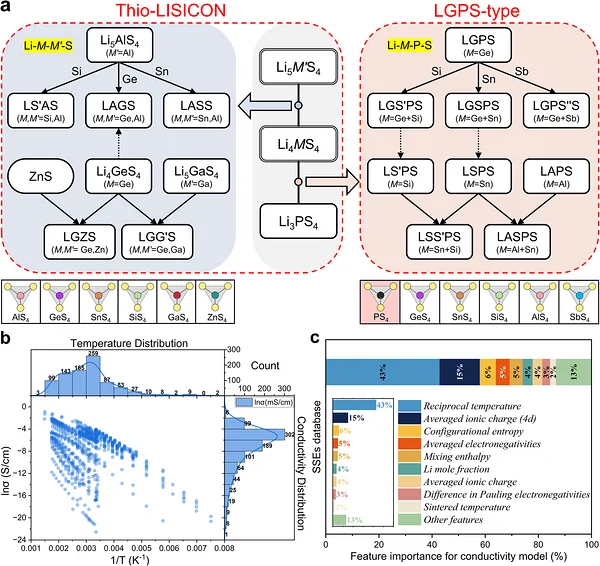
(a) The material design classification of SSEs with thio‐LISICON and LGPS‐type lattice structures. (b) Temperature and ionic conductivity distributions of 867 entries in the SSE database, highlighting the strong bias of the literature toward near‐ambient measurements and the resulting limitation in resolving non‐Arrhenius transport over broader temperature windows. (c) Significance of the feature attributes in the SSE database.

On this basis, a sulfide SSE database was curated containing 867 conductivity‐temperature entries from 87 literature sources, covering thio‐LISICON and LGPS‐type compositions across a broad range of transport behaviors (Data S1) [8, 20, 21, 23, 24, 25, 26, 27, 28, 29, 30, 31, 32, 33, 34, 35, 36, 37, 38, 39, 40, 41, 42, 43, 44, 45, 46, 47, 48, 49]. Because the database aggregates measurements reported under different synthesis histories, densification states, and impedance‐analysis practices, the extracted trends should be interpreted as chemically informed statistical regularities rather than strict intrinsic transport laws for every individual material. In addition, reported conductivities often include mixed grain‐ and grain‐boundary contributions, as these are not consistently deconvoluted in literature.

Figure 1b maps the conductivity‐temperature space covered by the dataset. The measurements span temperatures from −140°C to 400°C and conductivity values over many orders of magnitude but are strongly concentrated between −50°C and 100°C, reflecting the field's dominant emphasis on near‐ambient electrolyte benchmarking. By contrast, broader high‐temperature measurements remain comparatively sparse, even though deviations from ideal Arrhenius behavior often become more evident over extended temperature windows. The present literature landscape is therefore rich in conductivity benchmarks but comparatively poor in systematic information on transport nonlinearity.

These features of the literature motivate a systematic data‐driven analysis of sulfide SSE transport. Predictive modeling can, in principle, accelerate the exploration of superionic conductors, but two limitations are especially relevant here [58]. First, broad structural and compositional diversity can compromise model robustness and transferability if the chemical space is not carefully constrained [59]. By focusing on thio‐LISICON and LGPS‐type sulfides built from MS_4_ tetrahedra and excluding halide‐ and oxygen‐substituted out‐of‐domain chemistries [53], the present dataset preserves meaningful diversity while limiting confounding from fundamentally different framework topologies and bonding chemistries. Second, conductivity‐based learning provides limited chemical interpretability because ionic conductivity is strongly dominated by measured temperature. As shown in Figure 1c, test temperature accounts for the largest feature importance in conductivity‐based learning, indicating that the model predominantly captures the obvious thermal trend rather than subtler structure‐transport relationships encoded in lattice and compositional descriptors.

To improve chemical interpretability, physics‐informed descriptors were therefore constructed for the SSE database. Guided by prior machine‐learning studies of battery materials [60, 61, 62, 63, 64, 65, 66], 13 physicochemical quantities were first selected to represent key structural and ionic characteristics of sulfide SSEs, including averaged ionic charge and averaged ionic radius at the occupied 2*b* and 4*d* sites in LGPS‐type‐related frameworks (Table S1). These were expanded into 25 designed features for the final database (Table S2), and their pairwise correlations are summarized in Figure S1. Recursive feature elimination was then used to reduce redundancy and mitigate overfitting, followed by random‐forest‐based feature attribution analysis [67]. The three most influential variables in conductivity‐based learning are test temperature (43%), average ionic charge at the 4*d* site (15%), and configurational entropy (6%) (Figure 1c). This ranking is chemically reasonable [6, 68, 69], but it also underscores a central limitation of conductivity as the direct learning target: temperature dominates the variance, while descriptor‐level effects remain only partially resolved. Together, Figure 1b,c show that the current sulfide SSE literature is not only unevenly sampled in temperature but also poorly represented by conventional conductivity‐based targets when non‐Arrhenius transport is the phenomenon of interest. This motivates the introduction of a descriptor that retains temperature‐dependent transport variation in a comparable and chemically interpretable form.

### 2.2 Meyer–Neldel Deviation as a Quantitative Descriptor of Non‐Arrhenius Ion Transport

Analysis of the sulfide SSE database shows that more than 40% of the entries exhibit visible curvature in their Arrhenius plots beyond the near‐ambient temperature window (Figures S2 and S3), indicating that non‐Arrhenius transport is widespread but not systematically quantified across this materials class. When ionic conductivity is used directly as the prediction target, the learning problem becomes dominated by the strong Arrhenius‐type dependence on temperature. As shown in Figure 1c, measured temperature accounts for the largest fraction of model variance, so the model primarily captures the obvious thermal trend while subtler structure‐transport relationships remain partially obscured. Using a single temperature‐independent activation energy as the target has the opposite limitation: all σ(*T*) points for a given material collapse into one repeated label, and most of the information contained in the full conductivity‐temperature response is discarded. This redundancy severely limits the effective information content available for training and ignores non‐Arrhenius changes in *E*_a_ with temperature.

To overcome these limitations, a physics‐informed descriptor, termed MND, is introduced to quantify non‐Arrhenius ion transport. MND is conceptually distinct from conventional Meyer–Neldel analysis [52, 70]. Traditional Meyer–Neldel plots are generally constructed across a series of related materials to correlate activation energies and pre‐exponential factors and thereby reveal enthalpy‐entropy compensation. By contrast, the present framework evaluates the dispersion of apparent activation energies derived from multiple temperature‐pair fits within a single material relative to a global Arrhenius baseline. Each pairwise fit maps a conductivity‐temperature pair into the same (*E*_a_, lnσ_0_) parameter space used in Meyer–Neldel analysis: an ideal Arrhenius conductor would collapse to a single point in this space, whereas non‐Arrhenius transport produces a finite within‐material dispersion. This construction converts Arrhenius curvature from a qualitative observation into a comparable material‐level descriptor. The computational protocol is summarized schematically in Figure 2a and described in detail in the Supporting Information.

**FIGURE 2:**
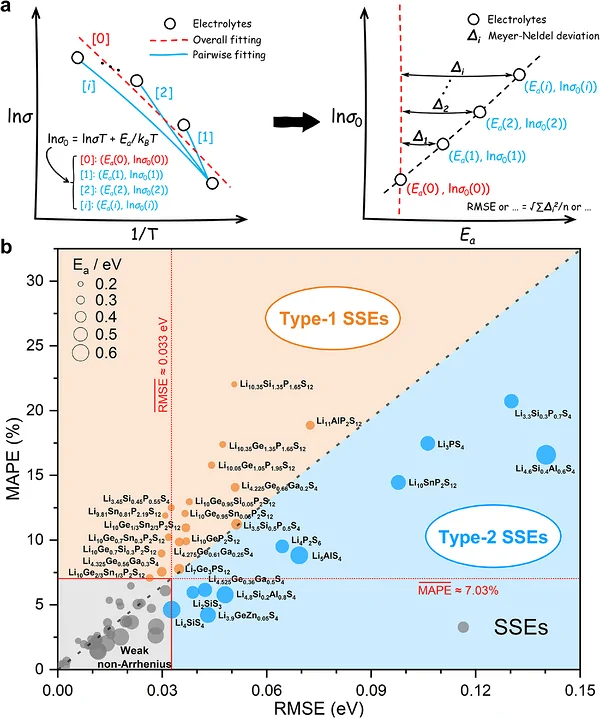
Conception and application of Meyer–Neldel deviation (MND). (a) Schematic definition of MND, in which the room‐temperature conductivity, or nearest available ambient‐temperature point, is used as a reference point to generate multiple pairwise apparent activation energies for each SSE relative to a global Arrhenius baseline. (b) RMSE‐MAPE landscape of MND across the sulfide SSE database, showing the operational separation of Type‐1 and Type‐2 materials according to the relative distribution of absolute and normalized activation‐energy dispersion.

For each SSE, the conductivity at room temperature, or at the nearest available ambient temperature, is selected as a common reference point. All remaining conductivity‐temperature data for that material are then paired with this reference to perform two‐point Arrhenius fits, generating a set of apparent (*E*_a_(*i*), *ln*σ_0_(*i*)) values for the same material. This choice standardizes the analysis across the literature because near‐ambient measurements are available for most entries and provide a consistent reference frame for pairwise comparison. The full conductivity‐temperature trace of each material is also fitted globally to obtain a baseline activation energy, *E*_a_(0). MND is then defined from the deviation of each pairwise apparent activation energy from this baseline value, thereby quantifying the extent to which the apparent migration barrier shifts with temperature. Meyer–Neldel plots generated from this procedure are shown in Figures S4 and S5. Because each material yields a set of deviations whose number depends on temperature coverage and sampling density, these deviations are aggregated into two complementary indicators, root‐mean‐square deviation (RMSE) and mean absolute percentage deviation (MAPE), as summarized in Figure S6 and discussed in detail in the Supporting Information. In short, RMSE captures the absolute magnitude of activation‐energy dispersion, whereas MAPE provides a normalized measure relative to the baseline activation energy. This formulation provides two immediate advantages. First, MND condenses the full temperature‐dependent variation of apparent activation energy in each SSE into comparable scalar descriptors, enabling direct ranking of compositions by their propensity for nonlinear Arrhenius behavior. Second, the set of MND‐derived apparent activation energies provides a temperature‐resolved and physics‐consistent target representation for subsequent supervised learning, preserving information that is lost in conventional single‐value *E*_a_ fitting.

Figure 2b maps the RMSE and MAPE values of MND across the sulfide SSE database. Each bubble represents one SSE, and the bubble size denotes the baseline activation energy, *E*_a_(0). Using the average of RMSE (∼0.033 eV) and MAPE (∼7.03%) over all samples as reference values, an operational partition of the RMSE‐MAPE space can be defined to compare the relative propensity for nonlinear Arrhenius behavior across the database. These dataset‐derived mean values are used solely as operational reference points for organizing the present distribution and should not be interpreted as universal physical thresholds for non‐Arrhenius transport. Materials lying below both reference values show comparatively small deviations from a linear Arrhenius description, whereas materials exceeding one or both reference values exhibit stronger departures from single‐barrier behavior. These deviations are consistent with genuine non‐Arrhenius transport behavior, although contributions from measurement uncertainty, impedance‐model choice, or microstructural evolution cannot be fully excluded. Notably, SSEs containing Si or Al often exhibit elevated RMSE or MAPE values, consistent with the increased structural complexity and phase heterogeneity frequently associated with these chemistries [24, 36, 46, 49].

Because MND is constructed from pairwise Arrhenius fits, the sensitivity of the resulting metrics to measurement uncertainty and analysis choices was examined explicitly. A temperature‐independent multiplicative offset in conductivity cancels in the logarithmic difference between a paired measurement and its reference, whereas point‐to‐point uncertainty propagates into the apparent activation energy with a magnitude that increases as |1/*T*_*i*_ − 1/*T*_0_| decreases. Consistent with this relation, numerical perturbation of the conductivity data by 1%–3% produced the largest variation for closely spaced temperature pairs, while exclusion of pairs with *ΔT* < 2 K suppressed this near‐reference amplification without materially changing the material‐level MND trends (Figure S7). Reference‐point sensitivity was further evaluated using the lowest‐, highest‐, and median‐temperature measurements together with a leave‐one‐out analysis. The corresponding Spearman correlations relative to the room‐temperature‐reference baseline ranged from 0.666 to 0.988 for RMSE and from 0.758 to 0.990 for MAPE, with classification consistencies of 75.8%–95.2% (Figure S8 and Table S3). The higher sensitivity observed for the highest‐ and median‐temperature references is expected when the selected reference point crosses distinct transport regimes, whereas the lowest‐temperature and leave‐one‐out analyses preserve the baseline ranking more closely. Finally, reducing the temperature‐sampling density to 5 and 10 K intervals retained RMSE/MAPE rank correlations of 0.903–0.989 and classification consistencies of 90.3%–95.2% (Table S4). Thus, the numerical value of MND is not strictly invariant to reference choice or sampling density, but the material‐level trends remain sufficiently stable under experimentally relevant perturbations to support comparative analysis across the database.

To further organize the RMSE‐MAPE landscape, a disproportion factor is defined as the ratio of the two average reference values, $k=\bar{MAPE}/\bar{RMSE}$ (black dashed line in Figure 2b). SSEs with *MAPE* < *k* · *RMSE* are labeled as Type‐1, whereas those with *MAPE* > *k* · *RMSE* are labeled Type‐2. Type‐1 SSEs, therefore, exhibit comparatively low MAPE but high RMSE and are generally associated with larger baseline activation energies. Conversely, Type‐2 SSEs show comparatively high MAPE but low RMSE and are generally associated with smaller baseline activation energies. Because MAPE is normalized by *E*_a_(0), this distinction reflects how absolute and relative barrier variations are distributed across materials with different activation‐energy scales. The Type‐1/Type‐2 separation should be viewed as an operational classification rather than a universal taxonomy. Its value depends on whether the resulting groups exhibit systematic differences in chemistry and structure, which are examined next through physicochemical descriptors.

### 2.3 Descriptor‐Level Classification of Type‐1 and Type‐2 SSEs

To determine whether the Type‐1 and Type‐2 classes identified in the RMSE‐MAPE landscape reflect systematic differences in chemistry and structure, the discriminative power of each descriptor was quantified using a combination of Z‐score normalization and relative error (Figure 3a). The Z‐score measures the deviation of a class‐specific mean from the global mean after normalization by the global standard deviation, whereas the relative error captures the magnitude and sign of the deviation from the global mean (formulas in the Supporting Information). Applying a Z‐score threshold above 0.8 and a relative‐error‐difference threshold above 50% identifies five key descriptors: the ionic radius mismatch *δ*, the difference in Pauling electronegativity Δ*χ*, the configurational entropy Δ*S*_conf_, the mixing enthalpy Δ*H*_mix_, and the mixing free energy Δ*G*_mix_, where Δ*G*_mix_ = Δ*H*_mix_  − *T*Δ*S*_conf_.

**FIGURE 3:**
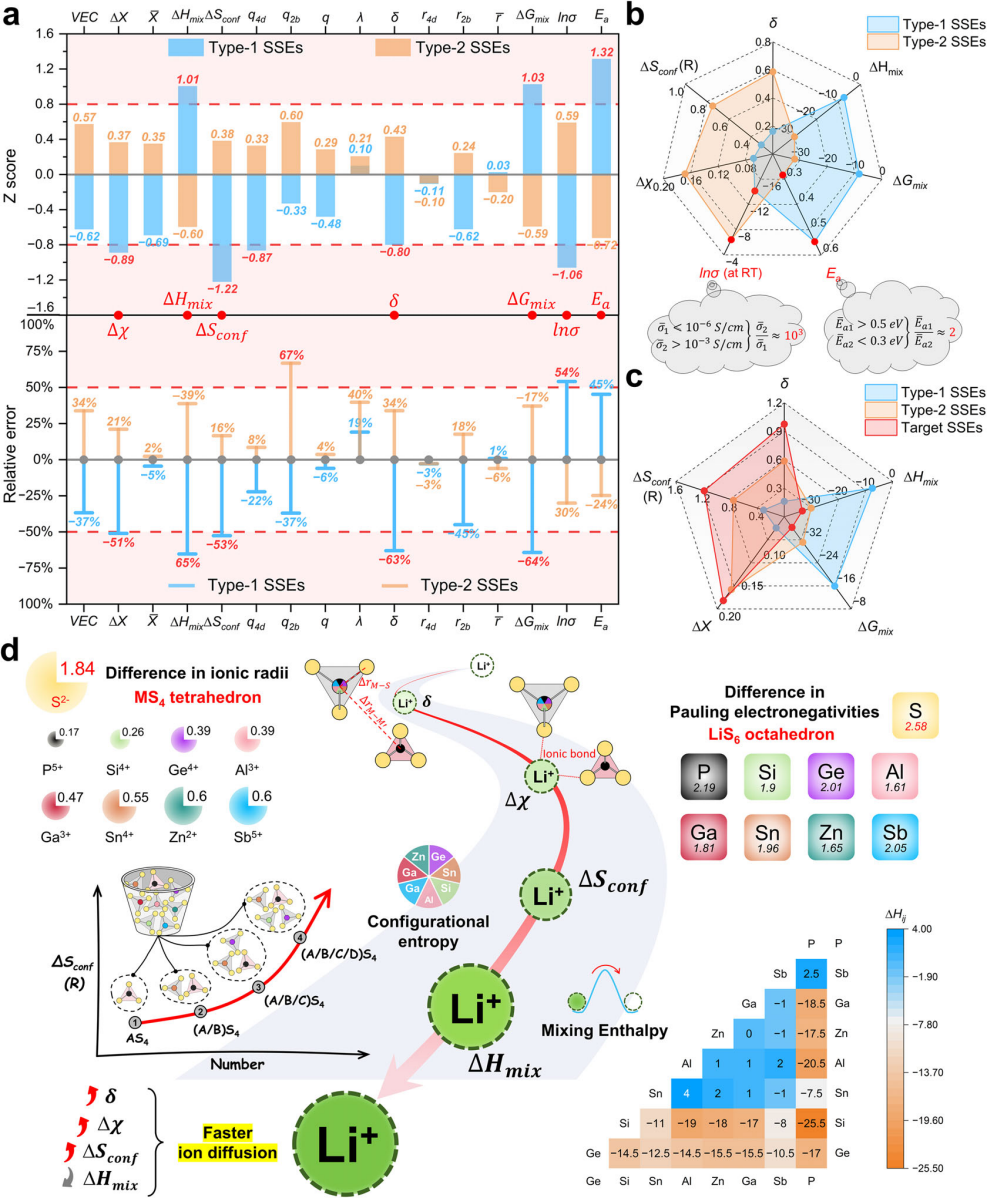
Descriptor‐based separation of Type‐1 and Type‐2 sulfide electrolytes. (a) Discriminative power of physicochemical descriptors evaluated by Z‐score and relative error. (b) Radar comparison of the key descriptors separating Type‐1 and Type‐2 classes. (c) Mapping of the two classes in descriptor space, highlighting the regime associated with high conductivity and low activation barrier. (d) Chemically grounded interpretation of how lattice mismatch, electronegativity contrast, configurational disorder, and mixing thermodynamics are associated with the observed transport‐class differences.

The descriptor contrast between the two classes is summarized in Figure 3b. Type‐1 SSEs are characterized by comparatively small *δ*, Δ*χ*, and Δ*S*_conf_ together with comparatively large Δ*H*_mix_ and Δ*G*_mix_. These materials correspondingly exhibit average room‐temperature ionic conductivities $\bar{\sigma }$ below 10^−6^ S cm^−1^ and activation energies $\bar{E_{a}}$ above 0.5 eV at room temperature. In contrast, Type‐2 SSEs display larger *δ*, Δ*χ*, and Δ*S_conf_* alongside smaller Δ*H*_mix_ and Δ*G*_mix_, and achieve $\bar{\sigma }$ above 10^−3^ S cm^−1^ at room temperature with $\bar{E_{a}}$ below 0.3 eV. Mapped back into descriptor space, these contrasts define a practically useful target regime of sulfide electrolytes, combining high conductivity and low activation barrier, which are preferentially associated with the Type‐2 descriptor pattern (Figure 3c).

Figure 3d provides a chemically grounded interpretation of why these descriptors separate the two regimes. A larger ionic‐radius mismatch *δ* is consistent with increased local geometric distortion of the MS_4_ tetrahedral framework and a broader distribution of Li coordination environments, which may increase the density of accessible hopping configurations and diffusion pathways [71]. Likewise, a larger electronegativity contrast Δ*χ* is often associated with a softer and more polarizable anion framework, a factor frequently linked to lower Li‐ion migration barriers [72]. A large configurational entropy Δ*S*_conf_ lowers the Gibbs free energy of mixing and can stabilize chemically disordered solid solutions, thereby favoring a more connected network of Li sites and transport pathways [68, 69]. Conversely, when a positive mixing enthalpy is insufficiently compensated by configurational entropy, the resulting increase in Δ*G*_mix_ is consistent with a greater tendency toward chemical segregation or secondary‐phase formation, both of which can disrupt fast‐ion percolation and lower measured conductivity [73]. These trends therefore suggest that non‐Arrhenius transport in sulfide electrolytes is systematically associated with lattice mismatch, framework polarizability, disorder, and mixing thermodynamics.

The RMSE‐MAPE map converts Arrhenius‐plot curvature into a directly comparable measure of nonlinearity, while the descriptor analysis links the resulting transport classes to chemically meaningful variables. These associations do not establish a universal microscopic mechanism for every individual SSE, but provide a chemical basis for identifying composition spaces likely to combine low activation barriers with comparatively controlled temperature‐dependent transport behavior over the intended operating window.

### 2.4 MND‐Based Learning of Non‐Arrhenius Ion Transport

Introducing MND not only quantifies non‐Arrhenius transport but also provides a more informative learning target for supervised modeling. Assigning a single temperature‐independent activation energy from a global Arrhenius fit has an intrinsic limitation, because all conductivity‐temperature records of a given material share the same label. In this representation, most of the information contained in the full σ(*T*) response is discarded, and the effective target variance available for learning is severely compressed. Consistent with this limitation, a random‐forest regressor trained on a single global *E*_a_(0) label shows only limited predictive performance under the present data partitioning scheme, as illustrated in Figure 4a. This result indicates that such a compressed target makes it difficult for the model to recover meaningful correlations between the static activation energy and the underlying physicochemical descriptors.

**FIGURE 4:**
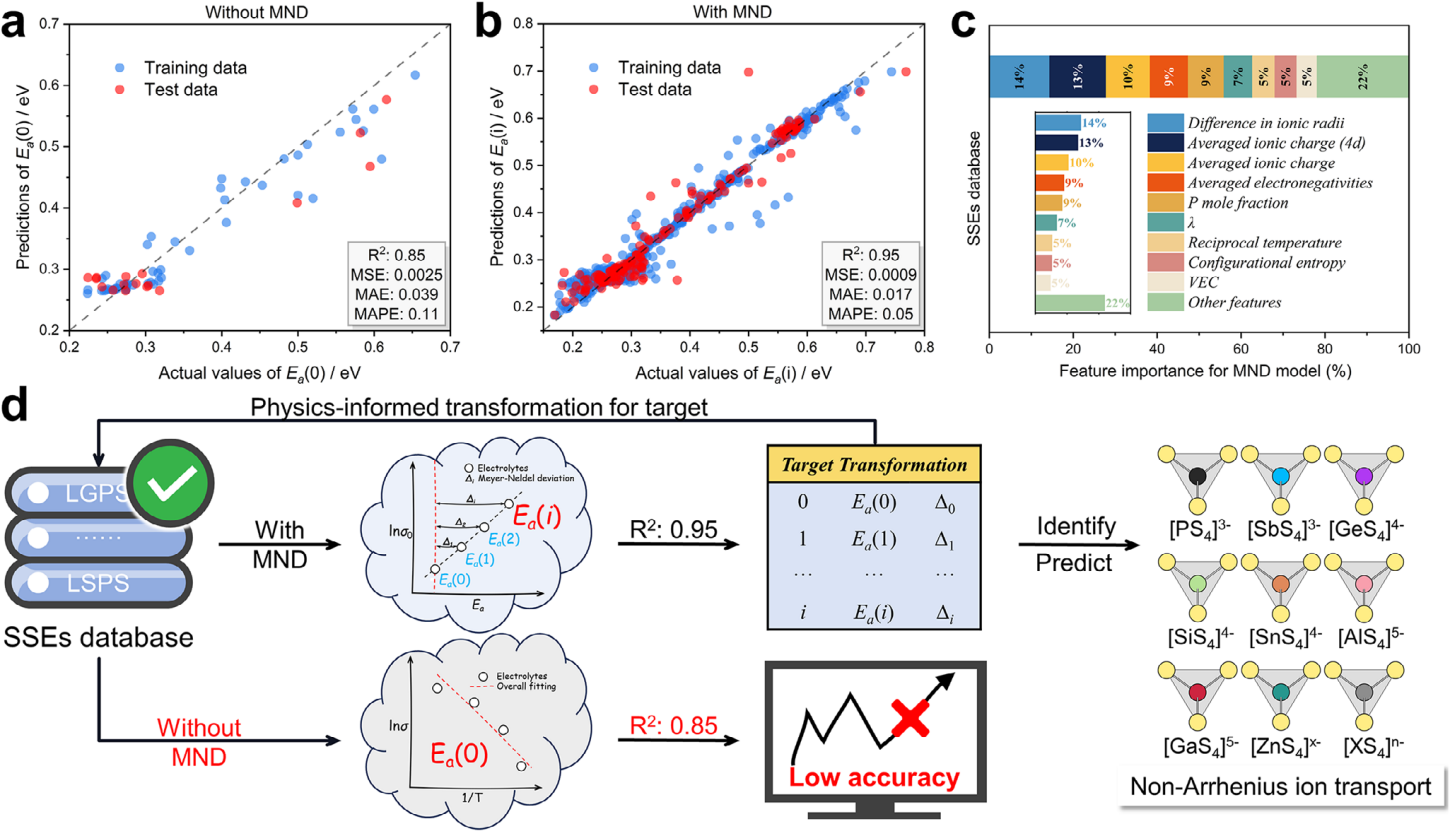
Physics‐informed learning enabled by the MND framework. (a) Limited predictive performance when training on single global activation energies that do not retain temperature dependence. (b) Improved performance when learning temperature‐resolved apparent activation energies generated through the MND framework. (c) Feature‐importance analysis for the MND‐based model, showing reduced temperature dominance and increased emphasis on chemically meaningful descriptors. (d) Schematic comparison between conventional single‐label learning and MND‐based temperature‐resolved target construction.

The MND framework addresses this limitation by redefining the target in a temperature‐resolved and physics‐consistent manner. Instead of assigning one *E*_a_(0) per material, pairwise Arrhenius fits relative to a common reference point generate a series of apparent activation energies, *E*_a_(*i*), that encode how the barrier evolves with temperature. Each conductivity‐temperature record thereby contributes a nonredundant MND‐consistent target, enriching the label space while preserving physical meaning. When the same random‐forest regressor is trained on this expanded set of *E*_a_(*i*), the test performance improves markedly to *R* ^2^ ≈ 0.95 with *MAE* ≈ 0.017 *eV* under the present dataset framework (Figure 4b). Independent benchmark models, including multilayer perceptron and XGBoost [74, 75], show the same qualitative trend, with XGBoost providing the best balance of accuracy and robustness within the present dataset framework (Figure S9 and Table S5).

Feature‐importance analysis further shows that MND‐based targets reduce temperature‐dominated learning and refocus the model on materials chemistry. In the conductivity‐prediction model, temperature alone accounts for about 43% of the variance (Figure 1c), indicating that conductivity is dominated primarily by the thermal trend rather than by descriptor‐level transport chemistry. By contrast, in the MND‐based *E*_a_(*i*) model, the most important variables are the difference in ionic radii (∼14%) and the averaged ionic charge at the 4d site (∼13%), whereas temperature is reduced to only ∼5% (Figure 4c). This more even distribution of feature importance indicates that trivial temperature effects no longer dominate the model and that descriptor‐level contributions to non‐Arrhenius transport can be recovered more effectively.

Figure 4d summarizes this physics‐informed learning strategy. By converting each conductivity trace into a series of MND‐derived activation‐energy targets, the framework replaces a single compressed label with a temperature‐resolved target representation that is both comparable and physically interpretable. In this way, MND provides a practical bridge between experimentally measured conductivity curves and descriptor‐based modeling, enabling non‐Arrhenius transport behavior to be learned in a form that is more directly useful for materials design within the present dataset representation.

### 2.5 Transferability of the MND Representation Across Diverse Solid‐Electrolyte Chemistries

The initial analysis focuses on the chemically constrained thio‐LISICON/LGPS‐type space to establish the MND framework without introducing additional variation from fundamentally different diffusion topologies. However, MND is defined solely from the temperature‐dependent conductivity response and therefore does not depend on sulfide‐specific structural descriptors. Its applicability beyond sulfide electrolytes was examined using the experimental ionic‐conductivity dataset reported by Pereznieto et al. [76], which originally contains 4826 conductivity‐temperature records from 166 solid‐electrolyte compositions. Because the source dataset was compiled for conductivity prediction rather than material‐resolved analysis of non‐Arrhenius transport, substantial preprocessing was required to standardize material identities, conductivity records, and temperature sampling, as described in the Supporting Information. The resulting dataset contains 160 compositions and 2410 conductivity‐temperature entries spanning seven solid‐electrolyte families, including garnet‐type, LISICON, argyrodite, perovskite‐type, and NASICON conductors. MND could be evaluated across these chemically and structurally distinct families, with mean RMSE and MAPE values of approximately 0.033 eV and 5.84%, respectively (Figure 5a). This broader analysis indicates that the MND representation is not intrinsically restricted to the original thio‐LISICON/LGPS‐type chemical space.

**FIGURE 5:**
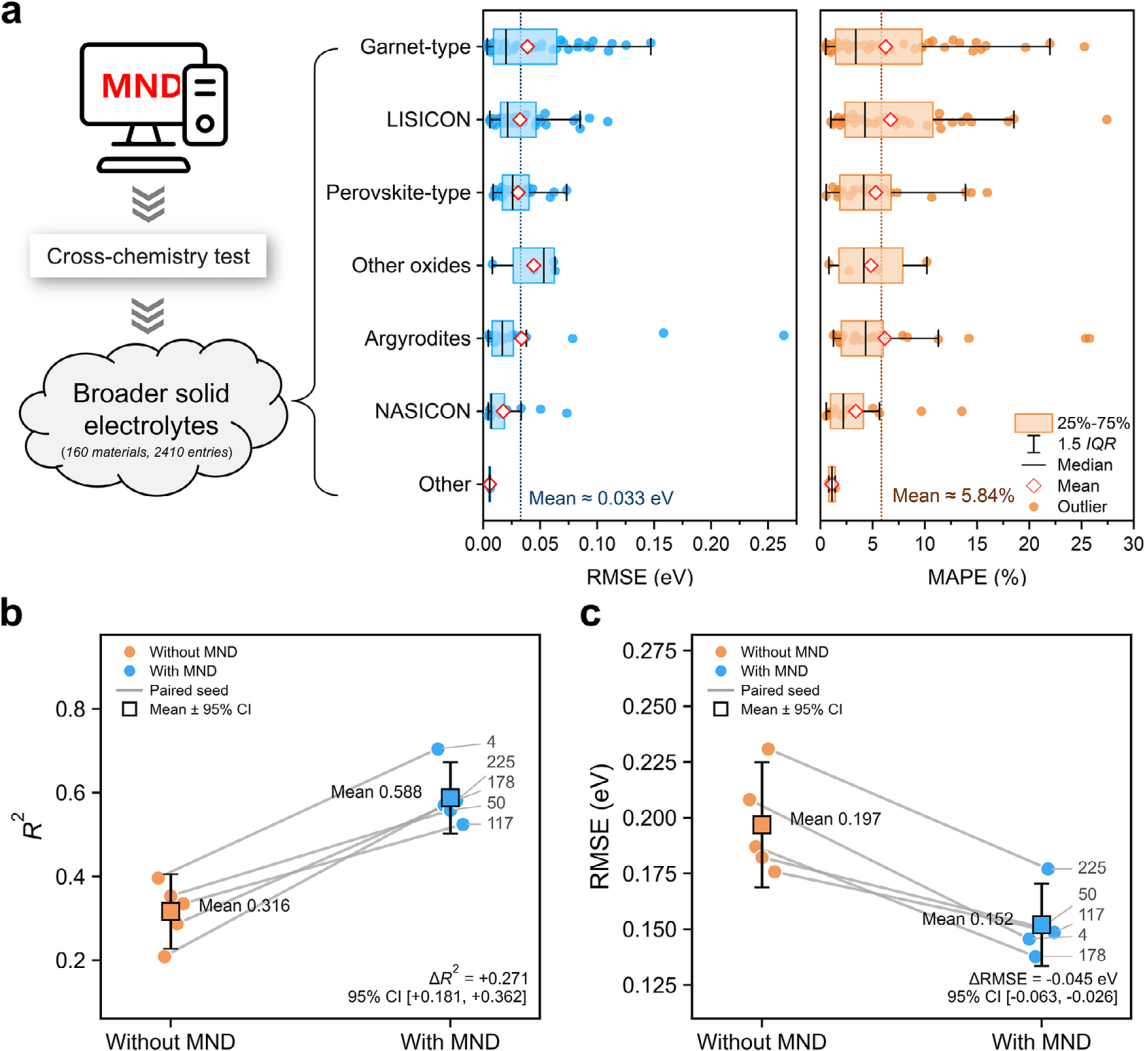
Cross‐chemistry transferability of the MND framework. (a) MND‐based RMSE and MAPE distributions across a broader solid‐electrolyte dataset comprising 160 compositions and 2410 conductivity–temperature entries from seven solid‐electrolyte families. Dotted lines indicate the dataset mean values. (b, c) Comparison of random‐forest performance across five repeated train/test partitions constructed by composition, using conventional global activation energy (Without MND) and temperature‐resolved apparent activation energy (With MND).

The broader dataset was used only to evaluate the cross‐chemistry transferability of the MND representation and was not incorporated into the subsequent LGSSPS screening. The feature space used for the original sulfide model contains structure‐informed descriptors specific to thio‐LISICON and LGPS‐type frameworks, including site‐resolved ionic properties associated with the *2b* and *4d* crystallographic sites. These descriptors are not directly defined across the structurally diverse electrolyte families represented in the broader dataset. The cross‐chemistry analysis therefore used the composition‐derived descriptors provided with the original literature dataset, whereas the subsequent LGSSPS screening remained based exclusively on the original sulfide dataset and its structure‐specific descriptors.

The predictive value of the temperature‐resolved MND representation was further examined in this more heterogeneous chemical space. Random‐forest models were evaluated using five repeated train/test partitions generated with different random seeds. Each partition was constructed at the composition level, ensuring that all records from a given composition were assigned exclusively to either the training or test set. The conventional global activation‐energy representation (without MND) and the MND‐derived temperature‐resolved representation (with MND) were evaluated on the same test compositions within each partition. Representative prediction results are shown in Figure S10. Across the five partitions, the MND representation increased the mean test *R* ^2^ from 0.316 to 0.588, corresponding to a mean within‐partition improvement of 0.271 with a 95% confidence interval of 0.181 to 0.362. The mean RMSE decreased from 0.197 to 0.152 eV, corresponding to a mean reduction of 0.045 eV with a 95% confidence interval of 0.026 to 0.063 eV (Figure 5b,c). MAE showed the same consistent improvement, decreasing from 0.137 to 0.114 eV, whereas the reduction in MAPE from 32.7% to 29.2% was less conclusive because the 95% confidence interval of the within‐partition difference included zero (Figure S11). The consistent improvement across composition‐level partitions indicates that the predictive benefit of the MND representation is retained across chemically distinct solid‐electrolyte families.

### 2.6 MND‐Guided Screening and Experimental Validation of LGPS‐Type Sulfides

The workflow shown in Figure 6a was applied to identify sulfide electrolytes that simultaneously exhibit a Type‐2 transport profile, characterized by high conductivity and low activation energy, and weak non‐Arrhenius behavior, quantified by low MND‐based RMSE and MAPE. The workflow integrates five elements, including (i) construction of a curated dataset of thio‐LISICON and LGPS‐type SSEs, each represented by a series of descriptors and experimentally measured transport properties; (ii) MND analysis of conductivity‐temperature traces to quantify non‐Arrhenius transport and extract temperature‐dependent activation energies *E*_a_(*T*), which in turn allows all entries to be grouped into Type‐1 (low σ, high *E*_a_) and Type‐2 (high σ, low *E*_a_) classes; (iii) training of machine‐learning models using MND‐derived targets to establish relationships between materials descriptors and transport behavior; (iv) application of these models to define a screening space for unexplored compositions that are predicted to combine a Type‐2 transport profile with weak non‐Arrhenius behavior; and (v) experimental verification through synthesis, impedance measurements, and MND evaluation of selected candidates.

**FIGURE 6:**
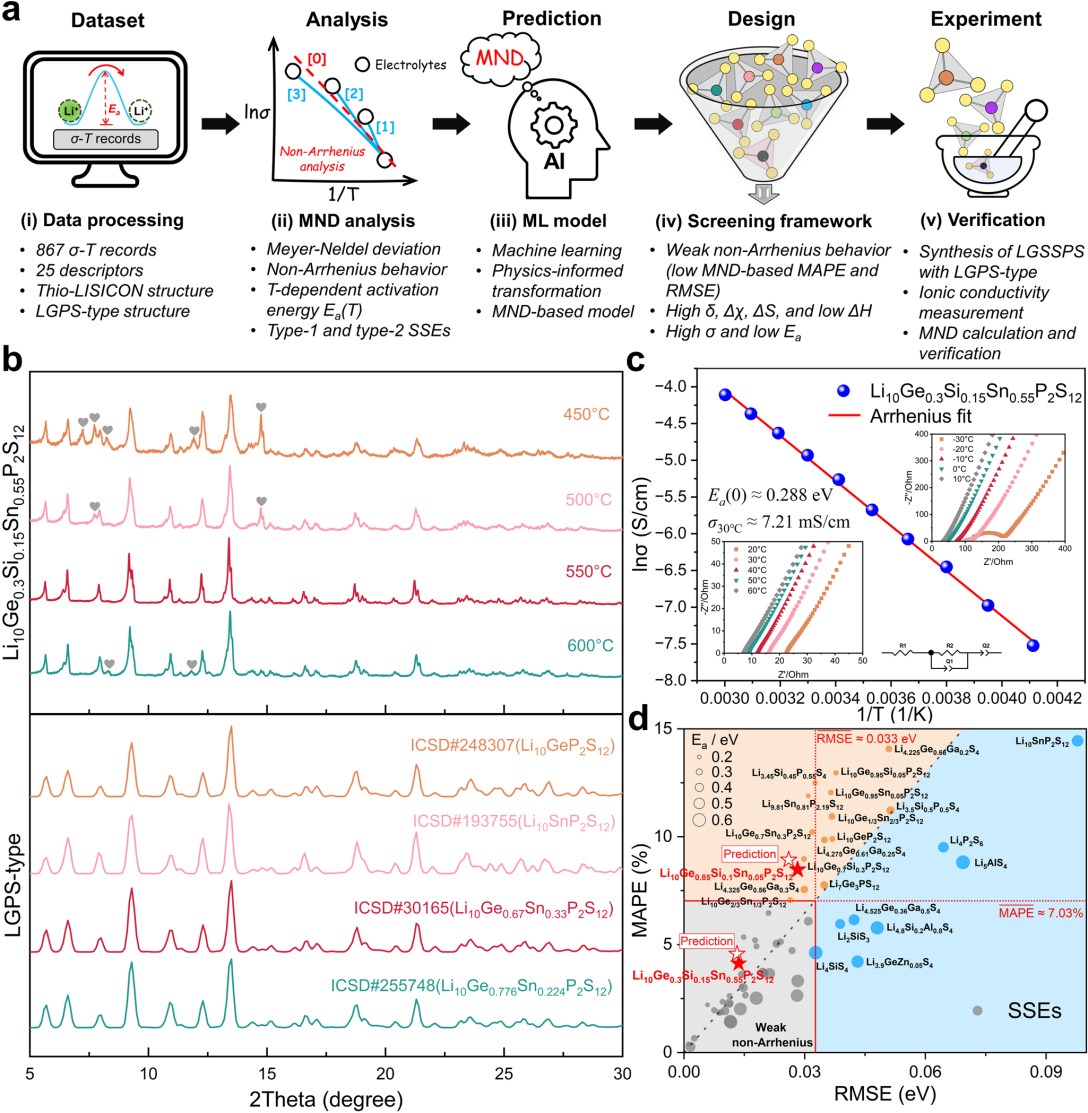
Prospective MND‐guided screening and experimental validation of LGSSPS. (a) Closed‐loop workflow integrating descriptor‐based Type‐2 screening, MND‐informed evaluation of non‐Arrhenius transport, and synthesis of prioritized candidates. (b) X‐ray diffraction patterns of Li_10_Ge_0.3_Si_0.15_Sn_0.55_P_2_S_12_ (LGSSPS) synthesized from 450°C to 600°C, compared with representative LGPS‐type phases; grey symbols mark impurity reflections. (c) Ionic conductivity, activation energy, and representative Nyquist plots of cold‐pressed LGSSPS (550°C) measured from −30°C to 60°C. (d) Comparison of predicted and experimental MND‐based RMSE and MAPE values for two selected compositions within the RMSE‐MAPE landscape of reported sulfide SSEs. Hollow and solid star markers denote predicted and experimental values, respectively.

Candidate compositions were then prioritized through sequential descriptor and MND‐based screening. The strategy was applied to the LGPS‐type compositional space Li_10_Ge*_x_* Si*_y_* Sn*_z_* P_2_S_12_, in which Ge^4+^, Si^4+^, Sn^4+^ and P^5+^ occupy tetrahedral framework sites. The compositional triangle defined by *x* + *y* + *z* = 1 was sampled at increments of 0.05, yielding 171 distinct compositions. Application of the five physicochemical criteria identified from the Type‐1/Type‐2 analysis retained 18 descriptor‐eligible compositions. These candidates were subsequently ranked using the predicted MND‐derived apparent activation energy at 30°C (Table S6), while the complete screening results for all 171 compositions are provided in Data S2. Prediction uncertainty was evaluated using residuals from the held‐out test set of the final random‐forest model. The model achieved a test *R* ^2^ of 0.952 and an RMSE of 0.030 eV. Calibration of an empirical 95% prediction interval gave a half‐width of approximately 0.063 eV. The resulting intervals overlap substantially among the closely ranked candidates and are therefore used to quantify prediction uncertainty rather than to imply statistically resolved differences between neighboring compositions.

Among the 18 descriptor‐eligible compositions, Li_10_Ge_0.3_Si_0.15_Sn_0.55_P_2_S_12_ (LGSSPS) ranked first according to the point prediction of the MND‐derived apparent activation energy at 30°C, with a predicted value of 0.261 eV and an empirical 95% prediction interval of 0.198 to 0.324 eV (Table S6). LGSSPS also falls within the Type‐2 region defined by the key discriminative descriptors (Figures 3c and S12), consistent with the targeted regime of high room‐temperature conductivity and low activation energy. This composition occupies a previously unreported region of the LGPS‐type compositional space, making it a useful experimental test of the screening strategy. The XGBoost model further predicts an MND‐based RMSE of approximately 0.013 eV and an MAPE of approximately 4.57%, indicating comparatively weak non‐Arrhenius behavior and motivating experimental validation.

To validate this prediction, Li_10_Ge_0.3_Si_0.15_Sn_0.55_P_2_S_12_ was synthesized and its structure and transport properties were examined. Figure 6b shows the x‐ray diffraction patterns of samples annealed from 450°C to 600°C. The sample treated at 550°C is consistent with the formation of the tetragonal LGPS‐type phase (space group *P4_2_/nmc*) and shows no discernible impurity reflections within the XRD detection limit. Electrochemical impedance spectroscopy on the 550°C sample (Figure 6c) yields a high room‐temperature ionic conductivity of 7.21 mS cm^−1^ in the cold‐pressed state. A classical Arrhenius fit gives an apparent activation energy of approximately 0.288 eV, consistent with the targeted high‐conductivity and low‐barrier regime. These measurements place Li_10_Ge_0.3_Si_0.15_Sn_0.55_P_2_S_12_ within the transport regime identified by the descriptor‐based screening.

Direct comparison of the predicted and experimental MND metrics provides a more stringent test of the screening result. Experimental analysis (Figure 6d) gives an MND‐based RMSE of approximately 0.014 eV and an MAPE of approximately 4.12%, in close agreement with the XGBoost predictions of approximately 0.013 eV and 4.57%, respectively. The predicted and experimental points occupy the same low‐deviation region of the RMSE‐MAPE landscape, indicating that Li_10_Ge_0.3_Si_0.15_Sn_0.55_P_2_S_12_ combines high ionic conductivity with the weak non‐Arrhenius behavior anticipated by the screening procedure. This agreement provides a proof of concept rather than evidence of universal predictive accuracy, showing that the combined descriptor and MND analysis can prioritize experimentally accessible sulfide compositions with favorable temperature‐dependent transport characteristics.

A second composition, Li_10_Ge_0.85_Si_0.1_Sn_0.05_P_2_S_12_, was examined as an additional control of the multistage screening procedure. This composition showed the lowest point‐predicted MND‐derived activation energy among all 171 enumerated compositions, but it was excluded from the descriptor‐eligible subset because it did not satisfy the electronegativity‐difference and ionic‐radius‐mismatch criteria. The major x‐ray diffraction reflections of the synthesized material are consistent with an LGPS‐type phase. Temperature‐dependent impedance measurements give an ionic conductivity of approximately 5.99 mS cm^−1^ at 30°C and a global apparent activation energy of approximately 0.281 eV (Figure S13). Its experimental MND‐based RMSE and MAPE are approximately 0.028 eV and 8.48%, respectively, in close agreement with the predicted values of 0.026 eV and 8.94%. These deviations are substantially larger than those of Li_10_Ge_0.3_Si_0.15_Sn_0.55_P_2_S_12_ (0.014 eV and 4.12%), despite the two compositions having comparable global apparent activation energies (0.281 versus 0.288 eV). Across the two experimentally examined compositions, the mean absolute relative deviations between predicted and experimental values are approximately 5.4% for MND‐based RMSE and 8.2% for MAPE. This comparison illustrates that a low single‐value activation energy alone does not ensure weak temperature‐dependent transport nonlinearity. The multistage screening strategy therefore aims to prioritize compositions that jointly combine high ionic conductivity, low activation barrier, and weak non‐Arrhenius behavior.

## 3 Conclusion

A curated database of SSEs was constructed to examine non‐Arrhenius ion transport within a chemically constrained thio‐LISICON/LGPS‐type materials space. Meyer–Neldel deviation (MND) is introduced as a quantitative descriptor that captures the temperature‐dependent dispersion of apparent activation energy and converts Arrhenius‐plot curvature into a comparable material‐level measure. More than 40% of the sulfide SSEs in the database exhibit notable deviations from linear Arrhenius behavior outside the near‐ambient regime, indicating that transport nonlinearity is widespread but remains insufficiently resolved in the current literature.

Mapping MND in RMSE‐MAPE space provides an operational classification of sulfide electrolytes into Type‐1 and Type‐2 transport regimes. These regimes are systematically associated with ionic‐radius mismatch, electronegativity difference, configurational entropy, and mixing thermodynamics, linking non‐Arrhenius transport behavior to chemically meaningful variables. MND also provides a more informative target representation for supervised learning than conventional single‐value activation‐energy labels by retaining temperature‐dependent transport information that would otherwise be lost. Its applicability is further supported by analysis of a broader dataset spanning seven solid‐electrolyte families, in which the MND representation consistently improves the prediction of activation‐energy behavior.

Descriptor and MND‐based screening of 171 Li_10_Ge*_x_* Si*_y_* Sn*_z_* P_2_S_12_ compositions identified Li_10_Ge_0.3_Si_0.15_Sn_0.55_P_2_S_12_ as the highest‐ranked descriptor‐eligible candidate by the MND‐derived point prediction. It exhibits a room‐temperature ionic conductivity of 7.21 mS cm^−1^, an apparent activation energy of approximately 0.288 eV, and weak non‐Arrhenius behavior in close agreement with prediction. A second experimentally examined composition, Li_10_Ge_0.85_Si_0.1_Sn_0.05_P_2_S_12_, provides an additional test of the multistage screening procedure. MND therefore provides a chemically interpretable representation for comparing temperature‐dependent ion transport and prioritizing sulfide compositions beyond conventional single‐value activation‐energy descriptions.

## Author Contributions

**Han Zhou**: conceptualization, writing – original draft, methodology, validation, investigation, visualization, data curation, formal analysis. **Xiaoxuan Chen**: conceptualization, methodology, writing – review and editing. **Hermann Tempel**: conceptualization, methodology, writing – review and editing. **Yong Yang**: conceptualization, methodology, writing – review and editing. **Boryann Liaw**: conceptualization, methodology, writing – review and editing, validation, visualization. **Shicheng Yu**: conceptualization, methodology, data curation, investigation, validation, formal analysis, supervision, funding acquisition, visualization, project administration, resources, writing – original draft, writing – review and editing. **Rüdiger‐A. Eichel**: supervision, resources, project administration, writing – review and editing.

## Conflicts of Interest

The authors declare no conflicts of interest.

## Supporting information




**Supporting File 1**: anie74900‐sup‐0001‐SuppMat.docx.

**Supporting File 2**: anie74900‐sup‐0002‐S1.xlsx.

**Supporting File 3**: anie74900‐sup‐0003‐S2.xlsx.

## Data Availability

The data that support the findings of this study are available in the Supporting Information and Supporting Data of this article. Additional processed data are available from the corresponding author upon reasonable request.
