# Supplementary material for: A Descriptor for Non‐Arrhenius Ion Transport Enables Design of Sulfide Superionic Conductors
Source: Angew Chem Int Ed Engl. 2026 Sep 19;65(45):e4940262. doi: 10.1002/anie.4940262 (PMC13725577; doi:10.1002/anie.4940262)
Supplement: Supplementary file 1 [file ANIE-65-e4940262-s003.docx]

Supporting Information

**A Descriptor for Non-Arrhenius Ion Transport Enables Design of Sulfide Superionic Conductors**

Han Zhou, Xiaoxuan Chen, Hermann Tempel, Yong Yang, Boryann Liaw, Shicheng Yu*, Rüdiger-A. Eichel

Materials and Methods

Supplementary Figure S1 to S13

Supplementary Tables S1 to S6

References [1] to [76]

^[1-20]^ ^[21]^ ^[22]^ ^[8, 20-21, 23-49]^ ^[50-52]^ ^[53]^ ^[54-57]^ ^[58]^ ^[59]^ ^[60-66]^ ^[67]^ ^[6, 68-69]^ ^[52, 70]^ ^[71]^ ^[72]^ ^[73]^ ^[74-76]^

**Materials and Methods**

**Experiment and Measurement**

The sulfide solid electrolyte Li_10_Ge_0.3_Si_0.15_Sn_0.55_P_2_S_12_ was synthesized via a conventional solid-state reaction route under an argon atmosphere. The starting materials used were Li_2_S (>99.9% purity, Alfa Aesar), P_2_S_5_ (>99% purity, Sigma Aldrich), GeS_2_ (>99.99% purity, VI HALBLEITERMATERIAL GmbH), SiS_2_ (>99.99% purity, VI HALBLEITERMATERIAL GmbH), SnS_2_ (>99.99% purity, VI HALBLEITERMATERIAL GmbH). These reagents were weighed in a molar ratio of Li_2_S:GeS_2_:SiS_2_:SnS_2_:P_2_S_5_ of 5:0.3:0.15:0.55:1, and the mixture was initially hand-milled for 30 minutes. The resulting mixture was subsequently subjected to planetary ball milling at 400 rpm for 12 h. The obtained powders were then thermally treated in a furnace at various target temperatures (450, 500, 550, 575, and 600 °C) for 30 h. After treatment, the furnace was allowed to cool naturally to room temperature. X-ray diffraction (XRD) patterns of the treated powder were recorded using an X-ray diffractometer (Panalytical Empyrean DY 1473) equipped with a Mo Kα radiation source (λ = 0.7107 Å). The transmission mode was used to obtain XRD data over a 2θ range of 5° to 55° at 55 kV and 40 mA, with three scans per sample. For ionic conductivity measurements, the treated powder was first pressed into a 10 mm diameter, 0.8-1 mm thick pellet at 500 MPa. AC impedance measurements were conducted with an excitation potential of 50 mV and a frequency range of 1 Hz to 7 MHz, using a potentiostat (VSP 300, BioLogic). The temperature was controlled between -30 °C and 60 °C using an environmental chamber (LabEvent T/20/40/EMC, Vötschtechnik). Li_10_Ge_0.85_Si_0.1_Sn_0.05_P_2_S_12_ was prepared using the same solid-state synthesis procedure. The precursor molar ratio of Li_2_S:GeS_2_:SiS_2_:SnS_2_:P_2_S_5_ was adjusted to 5:0.85:0.10:0.05:1, and the sample used for structural and transport characterization was annealed at 550 °C. XRD and temperature-dependent impedance measurements were performed under the same instrumental conditions described above.

**Description of the Sulfide Solid Electrolyte Database**

The database is compiled with a substantial collection of literature on SSEs from the past few decades, extracting data on synthesis temperatures, ionic conductivities, and activation energies reported in each study. A meticulous evaluation of the experimental procedures described in these publications has enabled us to understand how each sample was synthesized, characterized, and tested, ensuring that the data were clearly collected in each study. The ionic conductivity data were obtained from impedance spectroscopy measurements, where the values represent a mixture of grain and grain boundary contributions, as these are not always distinguished, and only a minority of studies provide sufficient detail to analyze the values accurately. Not limited to ionic conductivity at room temperature, the database comprehensively collects ionic conductivity for each material at various temperatures. This detailed collection facilitates a practical understanding of the relationship between temperature and ionic conductivity in subsequent Arrhenius analyses while maintaining data quality and integrity. Since most conductivity data in the literature are represented in Arrhenius plots, the extracted data typically include the logarithm of conductivity ($lg\sigma$, $ln\sigma$, $lg\sigma T$ or $ln\sigma T$) and the reciprocal temperature ($1/T$). Because literature Arrhenius plots employ different forms of conductivity normalization and logarithmic representation, the extracted data were converted to a consistent form before subsequent Arrhenius and MND analyses.

**Construction and Preprocessing of the Broader Solid-Electrolyte Dataset**

The broader dataset used to evaluate the cross-chemistry transferability of MND was derived from the experimental ionic-conductivity dataset reported by Pereznieto et al.,^[76]^ which originally contains 4826 conductivity-temperature records from 166 solid-electrolyte compositions. The source dataset was originally compiled for machine-learning prediction of ionic conductivity and was therefore reprocessed before MND analysis. Material identities, conductivity values, and measurement temperatures were standardized, and incomplete or unsuitable records were removed. To reduce the effect of uneven sampling, the experimentally reported point closest to 298.15 K was retained as the reference measurement for each composition. The remaining measurements were grouped on a 5 K temperature grid, and the experimentally measured point nearest to each grid value was retained. When an additional point fell within the same temperature interval as the reference measurement, the reference point was preserved. No conductivity values were generated by interpolation. Pairwise MND calculations were then performed relative to the experimentally observed near-room-temperature reference. Pairs with *ΔT* < 3 K were excluded, resulting in 2410 records from 160 compositions for the final MND analysis.

The final dataset spans seven solid-electrolyte families, including garnet-type, LISICON, argyrodite, perovskite-type, NASICON, other oxides, and other solid electrolytes. The composition-derived feature set provided with the original literature dataset was retained for the cross-chemistry machine-learning analysis. These descriptors were not regenerated in the present study. This representation differs from the structure-informed feature space used for the original sulfide solid-electrolyte dataset, which includes site-resolved ionic descriptors associated with the *2b* and *4d* crystallographic positions of thio-LISICON and LGPS-type frameworks. Such descriptors do not have a common structural definition across the broader set of electrolyte families. The broader dataset was therefore used exclusively to evaluate the cross-chemistry transferability of the MND representation and was not incorporated into the subsequent LGSSPS screening.

**Derivations and Discussion of Meyer-Neldel Deviation**

The Meyer-Neldel rule, also referred to as the isokinetic rule, describes a linear relationship between the logarithm of the pre-exponential factor (${ln\sigma}_{0}$) and the activation energy ($E_{a}$):

$$\begin{aligned} {ln\sigma}_{0}={ln\sigma}_{00}+\frac{E_{a}}{k_{B}T_{iso}}\#\left( S1 \right) \end{aligned}$$

where $\sigma_{00}$ serves as a process-dependent constant, while $T_{iso}$ denotes the isokinetic temperature, at which the conductivity becomes independent of the activation energy for a series obeying the Meyer-Neldel rule. The quantity $k_{B}T_{iso}$ is commonly referred to as the Meyer-Neldel energy. A conventional Meyer-Neldel plot therefore represents ${ln\sigma}_{0}$ as a function of $E_{a}$ across a series of related materials and is commonly associated with enthalpy-entropy compensation in thermally activated transport.^[70]^ Combining this relation with the Arrhenius expression used in the main text gives:

$$\begin{aligned} {ln\sigma}_{0}={\frac{1}{k_{B}T}\times E}_{a}+ln\sigma T\#\left( S2 \right) \end{aligned}$$

MND uses the same $\left( E_{a} , \ln\sigma_{0} \right)$ parameter space for a different purpose. For a given material, any two distinct conductivity-temperature measurements are sufficient to determine an apparent $E_{a}$ and $\ln\sigma_{0}$. In an ideal Arrhenius regime without measurement uncertainty, all temperature pairs from the same material yield identical values and therefore collapse to a single point in this parameter space. Non-Arrhenius transport instead produces temperature-dependent apparent activation energies and a finite within-material dispersion of the corresponding pairwise points. MND quantifies this dispersion rather than the conventional Meyer-Neldel correlation across different materials.

For each SSE, the room-temperature conductivity, or the measurement closest to room temperature when an exact room-temperature value is unavailable, is selected as the reference point. Each remaining temperature measurement is paired with this reference to obtain a set of apparent {$E_{a}(i)$, ${ln\sigma}_{0}(i)$} values. The near-room-temperature reference was selected because such measurements are available for most materials in the database and therefore provide a common experimental basis for comparison. Because all pairwise fits share the same reference point, the resulting {$E_{a}(i)$, ${ln\sigma}_{0}(i)$} values lie on a common reference line in Meyer-Neldel parameter space. This alignment is a consequence of the pairwise construction and is not itself interpreted as evidence of a conventional Meyer-Neldel relation. The sensitivity of MND to reference-point selection is examined separately in **Figure S8** and **Table S3**.

**Figure 2a** summarizes the computational procedure. All conductivity-temperature measurements for a given material are first fitted globally using the Arrhenius relation to obtain the baseline parameters $E_{a}(0)$ and ${ln\sigma}_{0}(0)$. The reference point is then paired with each remaining measurement to obtain a series of apparent activation energies $E_{a}(i)$ and pre-exponential factors ${ln\sigma}_{0}(i)$. The MND for each pair is defined as the deviation of the pairwise apparent activation energy from the global baseline:

$$\begin{aligned} \Delta_{i}=|E_{a}\left( i \right)-E_{a}\left( 0 \right)|\#\left( S3 \right) \end{aligned}$$

where $\Delta_{i}$ represents the absolute deviation of the pairwise apparent activation energy from the global Arrhenius value. For an ideal Arrhenius conductor, all pairwise fits yield the same activation energy, $E_{a}\left( i \right)=E_{a}\left( 0 \right)$, and therefore $\Delta_{i}=0$. Nonzero MND values indicate that apparent barriers deviate from the global Arrhenius value, whereas the magnitude of the deviation reflects the departure from a single temperature-independent activation energy, although contributions from experimental uncertainty cannot be excluded.

Because each material produces a set {$\Delta_{i}$} that depends on its temperature coverage and sampling density, direct comparison across materials requires aggregation to a single indicator. Therefore, analytic metrics such as Mean Squared Error (MSE), Root Mean Squared Error (RMSE), Mean Absolute Error (MAE), and Mean Absolute Percentage Error (MAPE) are utilized to summarize {$\Delta_{i}$} (**Figure S6**). The results indicate that MAE and RMSE show similar trends, while RMSE offers greater sensitivity to outliers due to the squaring of errors. Consequently, RMSE is chosen as the primary metric for assessing MND discrepancies across different SSEs:

$$\begin{aligned} RMSE=\sqrt{\frac{1}{n}\sum_{i=1}^{n} \Delta_{i}^{2}}\#\left( S4 \right) \end{aligned}$$

Meanwhile, MAPE offers a complementary perspective by expressing the error rate relative to the original activation energy $E_{a}(0)$, thereby more clearly distinguishing MND differences among SSEs:

$$\begin{aligned} MAPE=\frac{100\%}{n}\sum_{i=1}^{n} \frac{\Delta_{i}}{E_{a}(0)}\#\left( S5 \right) \end{aligned}$$

RMSE and MAPE are therefore used together to describe the absolute and relative magnitude of the activation-energy dispersion associated with non-Arrhenius ion transport.

**Robustness and Sensitivity Analysis of MND**

Because MND is derived from pairwise Arrhenius fits, the propagation of conductivity uncertainty into the apparent activation energy was examined explicitly. For a pair consisting of a reference point $\left( T_{0} , \sigma_{0} \right)$ and another measurement $\left( T_{i} , \sigma_{i} \right)$, the apparent activation energy is given by

$$\begin{aligned} E_{a}\left( i \right)=-k_{B}\frac{\ln\left( \sigma_{i}T_{i} \right)-ln(\sigma_{0}T_{0})}{1/T_{i}-1/T_{0}}\#\left( S6 \right) \end{aligned}$$

A temperature-independent multiplicative error in conductivity does not affect the pairwise activation energy. If all conductivity values are scaled by the same factor $C$,

$$\begin{aligned} \sigma_{i}^{'}=C\sigma_{i}\#\left( S7 \right) \end{aligned}$$

then

$$\begin{aligned} \ln\left( \sigma_{i}^{'}T_{i} \right)-ln(\sigma_{0}^{'}T_{0})=\ln\left( \sigma_{i}T_{i} \right)-ln(\sigma_{0}T_{0})\#\left( S8 \right) \end{aligned}$$

because the common $\ln C$ term cancels exactly. The global Arrhenius activation energy is likewise unchanged because the same scaling introduces only a constant vertical shift in the Arrhenius plot. Consequently, MND is invariant to a common multiplicative conductivity offset. Thus, any systematic scaling factor that remains constant across the measured temperature range does not propagate into the MND values.

In contrast, temperature-dependent or point-to-point errors do not cancel. More generally, if the two conductivity values are scaled by different factors $C_{i}$ and $C_{0}$, the resulting error in the pairwise activation energy is

$$\begin{aligned} E_{a}^{'}\left( i \right)-E_{a}\left( i \right)=-k_{B}\frac{\ln\left( C_{i}{/C}_{0} \right)}{1/T_{i}-1/T_{0}}\#\left( S9 \right) \end{aligned}$$

The propagated error therefore increases as $\left| 1/T_{i}-1/T_{0} \right|$ decreases, explaining the greater sensitivity of pairwise estimates for closely spaced temperature points. To emulate temperature-dependent point-to-point measurement uncertainty, each conductivity value, including the reference point, was independently perturbed using a mean-preserving lognormal multiplicative factor:

$$\begin{aligned} \sigma_{i}^{\left( r \right)}=\sigma_{i}\exp\left( \varepsilon_{i}^{\left( r \right)} \right), \varepsilon_{i}^{\left( r \right)}\sim N\left( -\frac{s^{2}}{2},s^{2} \right), s=\sqrt{ln(1+\eta^{2})}\#\left( S10 \right) \end{aligned}$$

where $\sigma_{i}^{\left( r \right)}$ is the perturbed conductivity of the $i$th temperature point in Monte Carlo realization $r$, and $\eta$ is the prescribed coefficient of variation, set to 1%, 2%, or 3%. The term $-s^{2}/2$ ensures that the multiplicative perturbation has unit mean and therefore does not introduce a systematic shift in conductivity. For each error level, 5000 Monte Carlo realizations were generated. Within each realization, the same perturbed reference point was used for all temperature pairs of a given material, and the global Arrhenius fit and all pairwise apparent activation energies were recalculated from the same perturbed conductivity curve. The resulting MND-based RMSE and MAPE values were evaluated using all temperature pairs and after excluding pairs with *ΔT* < 2 K (**Figure S7**). The perturbation analysis confirms that the largest variations occur for closely spaced temperature pairs, while excluding pairs with *ΔT* < 2 K substantially suppresses this near-reference amplification without materially altering the overall material-level MND trends.

Reference-point sensitivity was examined by repeating the MND calculation using the lowest-temperature, highest-temperature, median-temperature, and leave-one-out reference schemes. The resulting material-level RMSE and MAPE values were compared with the room-temperature-reference baseline using Spearman rank correlations, and the consistency of the Type-1/Type-2 classification was evaluated (**Figure S8** and **Table S3**). The numerical magnitude of MND therefore depends to some extent on the selected reference point, but the material-level trends remain correlated across the tested conditions.

The influence of temperature-sampling density was further evaluated by comparing the full dataset with datasets sampled at approximately 5 K and 10 K intervals. RMSE and MAPE rank correlations were calculated relative to the full-data baseline, and the operational classification thresholds were recalculated independently for each sampling scheme before evaluating Type-1/Type-2 consistency (**Table S4**). The retained correlations and classification consistency indicate that the material-level MND trends are reasonably robust to moderate variations in temperature-sampling density.

**Machine Learning Approach**

Machine-learning evaluations were performed using material-level data partitioning. For the original sulfide dataset, the dataset was split into training (80%) and test (20%) sets. Hyperparameter optimization was performed by five-fold cross-validation within the training set: four for model training and one for validation. All machine learning models were trained using the scikit-learn and XGBoost packages in Python, specifically leveraging random forests (RF), XGBoost, and the multi-layer perceptron (MLP) algorithm.^[67, 74-75]^ During the ML analysis, the primary purpose is to compare the informativeness of different target representations within a common dataset framework.

For the broader solid-electrolyte dataset, the same separation principle was retained. Five repeated train/test partitions were generated using different random seeds, with 75% of the compositions assigned to training and 25% to testing in each partition. Given the greater chemical heterogeneity of the broader dataset, repeated composition-level partitions were used to reduce the dependence of the transferability assessment on any single train/test assignment and to evaluate the consistency of the observed performance differences across different held-out composition sets. The conventional global activation-energy representation and the MND-derived temperature-resolved representation were evaluated on identical training and test compositions within each partition.

The Mean Absolute Error quantifies the average absolute difference between predicted and actual values, with lower MAE indicating better predictive accuracy. For the activation energy and ionic conductivity predictions in this study, the units of MAE are eV and mS/cm, respectively. The MAE is defined by:

$$\begin{aligned} MAE=\frac{1}{n}\sum_{i=1}^{n} \left| y_{i}-y_{i}^{'} \right|\#\left( S11 \right) \end{aligned}$$

where *y_i_* is the actual value, *y_i_*' is the predicted value, and *n* is the sample size.

The Mean Squared Error is another model assessment metric that measures the average of the squares of the differences between predicted and actual values. A lower MSE indicates that the model's predictions are more closely aligned with the actual data, which is desirable for a good model fit. MSE is computed using the formula:

$$\begin{aligned} MSE=\frac{1}{n}\sum_{i=1}^{n} \left( y_{i}-y_{i}^{'} \right)^{2}\#\left( S12 \right) \end{aligned}$$

where *y_i_* is the actual value, *y_i_*' is the predicted value, and *n* is the sample size.

**Prediction Uncertainty for Candidate Screening**

Prediction uncertainty for the 171 Li_10_Ge*_x_*Si*_y_*Sn*_z_*P_2_S_12_ compositions was estimated from the absolute residuals of the held-out data used with the final random-forest model. The model achieved *R*^2^ = 0.952, RMSE = 0.030 eV, MAE = 0.017 eV, and MAPE = 4.83% on the held-out set. An empirical 95% prediction interval was constructed using the finite-sample residual quantile

$$\begin{aligned} q_{0.95}=r_{\left( k \right)}, k=\left\lceil(n+1)(1-\alpha) \right\rceil\#\left( S13 \right) \end{aligned}$$

where $r_{\left( k \right)}$ is the $k$th ordered absolute residual, $n$ is the number of held-out observations, and $\alpha=0.05$. For $n=173$, $k=166$, giving $q_{0.95}=0.0634$eV. The empirical prediction interval for each candidate was therefore defined as

$$\begin{aligned} \left[ \hat{E_{a}}-q_{0.95}, \hat{E_{a}}+q_{0.95} \right]\#\left( S14 \right) \end{aligned}$$

The interval represents uncertainty associated with an individual candidate prediction rather than a confidence interval for the mean prediction. The original point predictions were retained for candidate ranking, and the uncertainty analysis therefore does not alter the archived ranking of the 171 compositions. Complete predictions, screening criteria, rankings, and empirical prediction intervals are provided in **Data S2**, while the 18 descriptor-eligible candidates are summarized in **Table S6**.

**Statistical Analysis Methods for Classification**

The Z-score in this work quantifies the deviation of each feature for Type-1 or Type-2 SSEs from the corresponding feature for the entire SSEs, normalized by the standard deviation of that feature across the entire SSEs. This adaptation allows systematic identification of features that most significantly differentiate the two SSE classes. The modified Z-score calculation proceeds as follows:

$$\begin{aligned} Z_{i}=\frac{X_{i}-\mu_{total}}{s_{total}}\#\left( S15 \right) \end{aligned}$$

where $X_{i}$ represents the average value of each feature for Type-1 or Type-2 SSEs. $\mu_{total}$ and $s_{total}$ represent the average value and standard deviation of each feature for all SSEs, respectively.

The relative error in this work is defined to quantify the directional deviation of each feature for Type-1 or Type-2 SSEs relative to the corresponding feature of the entire SSEs. This modified metric not only measures the magnitude of deviation but also retains polarity to indicate whether a feature is over (positive) or under (negative) the average value for the entire SSEs. The calculation proceeds as follows:

$$\begin{aligned} Relative error=\frac{\mu_{i}-\mu_{total}}{\left| \mu_{total} \right|}\#\left( S16 \right) \end{aligned}$$

where $\mu_{i}$ and $\mu_{total}$ represent the average values of each feature for Type-1 or Type-2 SSEs and all SSEs, respectively.

**Supplementary Figures**

**Figure S1**. Feature correlation analysis of sulfide solid electrolyte database.

**Figure S2.** Arrhenius plots for all sulfide solid electrolyte entries in the database.

**Figure S3.** Individual Arrhenius plots for the sulfide solid electrolytes in the database.

**Figure S4.** Meyer-Neldel plots and deviations of the SSEs from the database. The room temperature (or the closest available temperature) and the ionic conductivity of each SSE are chosen as the reference point.

**Figure S5.** Meyer-Neldel plots and deviations for each sulfide solid electrolyte.

**Figure S6.** MSE, RMSE, MAE and MAPE of Meyer-Neldel deviation for SSEs.

**Figure S7.** Sensitivity of MND to conductivity uncertainty and temperature separation. (a,b) Changes in MND-based RMSE and MAPE under independent point-to-point multiplicative conductivity perturbations of 1–3%, comparing all pairwise estimates with results obtained after excluding pairs with Δ*T* < 2 K. The dashed lines indicate the unperturbed values. (c) Propagated uncertainty in pairwise apparent activation energy as a function of reciprocal-temperature separation from the reference point.

**Figure S8.** Sensitivity of MND metrics and classification to reference-point selection. (a,b) Comparison of material-level MND-based RMSE and MAPE obtained using the lowest-temperature, highest-temperature, median-temperature, and leave-one-out reference schemes against the room-temperature reference baseline. Spearman correlation coefficients are indicated in each panel. (c) Classification consistency relative to the room-temperature baseline for the four alternative reference schemes.

**Figure S9.** Benchmark results in predicting $E_{a}(i)$ of SSEs with (a) XGBoost and (b) MLP models.

**Figure S10.** Representative prediction results in the broader solid-electrolyte dataset with and without the MND representation. Predicted versus actual activation energies for a representative composition-level train/test partition using (a) conventional global activation energy, $E_{a}(0)$, without MND and (b) temperature-resolved apparent activation energy, $E_{a}(i)$, with MND. Training and test data are shown separately, and the dashed lines indicate ideal predictions.

**Figure S11.** Paired comparisons of test (a) MAPE and (b) MAE across five composition-level train/test partitions. Grey lines connect results obtained from identical held-out composition sets. Squares and error bars indicate the mean and 95% confidence interval across the five splits.

**Figure S12.** Calculation and prediction of LGSSPS with all properties according to Type-1 and Type-2 SSEs.

**Figure S13.** Structural and temperature-dependent ionic-transport characterization of Li_10_Ge_0.85_Si_0.1_Sn_0.05_P_2_S_12_. (a) X-ray diffraction pattern of the sample annealed at 550 °C, compared with the reference LGPS-type Li_10_Ge_0.67_Sn_0.33_P_2_S_12_ phase (ICSD #30165). (b) Temperature-dependent ionic conductivity and Arrhenius analysis from −30 to 60 °C, yielding an apparent activation energy of approximately 0.281 eV and an ionic conductivity of approximately 5.99 mS cm^-1^ at 30 °C. Representative impedance spectra and the equivalent circuit used for fitting are shown in the insets.

**Supplementary Tables**

**Table S1.** The list of physicochemical features adopted in the SSE database.

| **Feature** | **Description** |
| --- | --- |
| $VEC=\sum_{i=1}^{n} C_{i}{(VEC)}_{i}$ | Valence electron concentration |
| $\Delta\chi=\sqrt{\sum_{i=1}^{n} C_{i}(\chi_{i}-\bar{\chi})}$ | Difference in Pauling electronegativities |
| $\bar{\chi}=\sum_{i=1}^{n} C_{i}\chi_{i}$ | Averaged electronegativities |
| $\Delta H_{mix}=\sum_{i=1,i\neq j}^{n} {4H}_{ij}C_{i}C_{j}$ | Mixing enthalpy |
| $\Delta S_{conf}=-R\sum_{i=1}^{n} (C_{i}\ln C_{i})$ | Configurational entropy |
| $q_{4d}=\sum_{i=1}^{n} C_{i}q_{{4d}_{i}}$ | Averaged ionic charge in 4*d* site of tetrahedra |
| $q_{2b}=\sum_{i=1}^{n} C_{i}q_{{2b}_{i}}$ | Averaged ionic charge in 2*b* site of tetrahedra |
| $\bar{q}=\sum_{i=1}^{n} C_{i}q_{i}$ | Averaged ionic charge |
| $\lambda=\frac{\Delta S_{config}}{\delta^{2}}$ | A geometrical parameter |
| $\delta=\sqrt{\sum_{i=1}^{n} {C_{i}(1-\frac{r_{i}}{\bar{r}})}^{2}}$ | Difference in ionic radii |
| $r_{4d}=\sum_{i=1}^{n} C_{i}r_{{4d}_{i}}$ | Averaged ionic radius in 4*d* site of tetrahedra |
| $r_{2b}=\sum_{i=1}^{n} C_{i}r_{{2b}_{i}}$ | Averaged ionic radius in 2*b* site of tetrahedra |
| $\bar{r}=\sum_{i=1}^{n} C_{i}r_{i}$ | Averaged ionic radius |

**Table S2.** The list of all features in the SSE database.

| **Category** | **Feature** | **Category** | **Feature** |
| --- | --- | --- | --- |
| Physicochemical^a^ | VEC | Composition | Li mole fraction |
|  | Difference in Pauling electronegativities |  | Ge mole fraction |
|  | Averaged electronegativities |  | Si mole fraction |
|  | Mixing enthalpy |  | Sn mole fraction |
|  | Configurational entropy |  | P mole fraction |
|  | Averaged ionic charge (4*d*) |  | Sb mole fraction |
|  | Averaged ionic charge (2*b*) |  | Zn mole fraction |
|  | Averaged ionic charge |  | Ga mole fraction |
|  | λ |  | Al mole fraction |
|  | Difference in ionic radii |  | S mole fraction |
|  | Averaged ionic radius (4*d*) | Synthesis | Sintering temperature |
|  | Averaged ionic radius (2*b*) |  | Measured temperature |
|  | Averaged ionic radius |  | - |

**Table S3.** Sensitivity of MND metrics and SSE classification to reference-temperature choice.

| Reference scheme | Mean RMSE | Mean MAPE | RMSE Spearman | MAPE Spearman | Classification consistency |
| --- | --- | --- | --- | --- | --- |
| Room-temperature baseline | 0.03267 | 7.026 | 1.000 | 1.000 | 100.0% |
| Lowest temperature | 0.03098 | 6.675 | 0.913 | 0.926 | 93.5% |
| Highest temperature | 0.03758 | 8.226 | 0.704 | 0.799 | 75.8% |
| Median temperature | 0.04308 | 8.665 | 0.666 | 0.758 | 83.9% |
| Leave-one-out | 0.03308 | 7.069 | 0.988 | 0.990 | 95.2% |

**Table S4.** Sensitivity of MND metrics to temperature-sampling density.

| **Sampling scheme** | **Pairwise points** | **RMSE Spearman** | **MAPE Spearman** | **Classification consistency** |
| --- | --- | --- | --- | --- |
| Full data | 570 | 1.000 | 1.000 | 100.0% |
| 5 K sampling | 558 | 0.963 | 0.989 | 95.2% |
| 10 K sampling | 516 | 0.903 | 0.940 | 90.3% |

**Table S5.** Performance of the different machine-learning models.

| **Benchmark** | **RF** | | | **XGBoost** | | | | **ANN** | | | |
| --- | --- | --- | --- | --- | --- | --- | --- | --- | --- | --- | --- |
|  | **Train** | **Test** | | **Train** | | **Test** | | **Train** | | | **Test** |
| *R*^2^ | 0.9723 | | 0.9475 | | 0.9680 | | 0.9500 | | 0.9433 | 0.9434 | |
| MSE | 0.0004703 | | 0.0008732 | | 0.0005439 | | 0.0008323 | | 0.0009624 | 0.0009430 | |
| MAE | 0.01254 | | 0.01691 | | 0.01404 | | 0.01732 | | 0.01920 | 0.01927 | |
| MAPE | 0.04098 | | 0.05317 | | 0.04294 | | 0.05280 | | 0.05602 | 0.05597 | |

**Table S6.** Descriptor-eligible Li_10_Ge_x_Si_y_Sn_z_P_2_S_12_ candidates prioritized for MND-guided screening. The 18 compositions satisfying all first-stage physicochemical-descriptor criteria are ranked according to the predicted MND-derived apparent activation energy at 30 °C. Empirical 95% prediction intervals were calibrated from absolute residuals of the held-out test set. Complete screening results for all 171 compositions are provided in **Data S2**.

| Rank | Composition | Predicted MND-*E_a_* at 30 °C (eV) | Empirical 95% PI lower | Empirical 95% PI upper |
| --- | --- | --- | --- | --- |
| 1 | Li_10_Ge_0.3_Si_0.15_Sn_0.55_P_2_S_12_ | 0.2610 | 0.1976 | 0.3244 |
| 2 | Li_10_Ge_0.25_Si_0.2_Sn_0.55_P_2_S_12_ | 0.2614 | 0.1981 | 0.3248 |
| 3 | Li_10_Ge_0.2_Si_0.25_Sn_0.55_P_2_S_12_ | 0.2620 | 0.1986 | 0.3254 |
| 4 | Li_10_Ge_0.35_Si_0.1_Sn_0.55_P_2_S_12_ | 0.2630 | 0.1997 | 0.3264 |
| 5 | Li_10_Ge_0.4_Si_0.05_Sn_0.55_P_2_S_12_ | 0.2630 | 0.1997 | 0.3264 |
| 6 | Li_10_Ge_0.3_Si_0.1_Sn_0.6_P_2_S_12_ | 0.2633 | 0.1999 | 0.3267 |
| 7 | Li_10_Ge_0.25_Si_0.15_Sn_0.6_P_2_S_12_ | 0.2634 | 0.2000 | 0.3267 |
| 8 | Li_10_Ge_0.2_Si_0.15_Sn_0.65_P_2_S_12_ | 0.2639 | 0.2005 | 0.3273 |
| 9 | Li_10_Ge_0.2_Si_0.2_Sn_0.6_P_2_S_12_ | 0.2643 | 0.2009 | 0.3277 |
| 10 | Li_10_Ge_0.15_Si_0.3_Sn_0.55_P_2_S_12_ | 0.2660 | 0.2026 | 0.3294 |
| 11 | Li_10_Ge_0.15_Si_0.2_Sn_0.65_P_2_S_12_ | 0.2665 | 0.2032 | 0.3299 |
| 12 | Li_10_Ge_0.1_Si_0.3_Sn_0.6_P_2_S_12_ | 0.2671 | 0.2038 | 0.3305 |
| 13 | Li_10_Ge_0.05_Si_0.4_Sn_0.55_P_2_S_12_ | 0.2673 | 0.2039 | 0.3307 |
| 14 | Li_10_Ge_0.15_Si_0.25_Sn_0.6_P_2_S_12_ | 0.2675 | 0.2041 | 0.3309 |
| 15 | Li_10_Ge_0.1_Si_0.35_Sn_0.55_P_2_S_12_ | 0.2676 | 0.2042 | 0.3309 |
| 16 | Li_10_Ge_0.05_Si_0.35_Sn_0.6_P_2_S_12_ | 0.2683 | 0.2050 | 0.3317 |
| 17 | Li_10_Ge_0.1_Si_0.25_Sn_0.65_P_2_S_12_ | 0.2710 | 0.2077 | 0.3344 |
| 18 | Li_10_Ge_0.05_Si_0.3_Sn_0.65_P_2_S_12_ | 0.2725 | 0.2091 | 0.3358 |

*****Li_10_Ge_0.85_Si_0.1_Sn_0.05_P_2_S_12_ showed the lowest point-predicted MND-derived activation energy among all 171 compositions but was not included in the descriptor-eligible subset because it did not satisfy the electronegativity-difference and ionic-radius-mismatch criteria.

References

[1]	Y. Zeng, B. Ouyang, J. Liu, Y. W. Byeon, Z. Cai, L. J. Miara, Y. Wang, G. Ceder, *Science* **2022**, *378*, 1320-1324.

[2]	L. Zhu, Y. Wang, J. Chen, W. Li, T. Wang, J. Wu, S. Han, Y. Xia, Y. Wu, M. Wu, F. Wang, Y. Zheng, L. Peng, J. Liu, L. Chen, W. Tang, *Science Advances* **2022**, *8*, eabj7698.

[3]	P. Lu, S. Gong, F. Guo, X. Zhu, Y. Huang, Y. Wang, W. He, M. Yang, L. Chen, H. Li, F. Wu, *Nano Energy* **2023**, *118*, 109029.

[4]	J. Sung, J. Heo, D.-H. Kim, S. Jo, Y.-C. Ha, D. Kim, S. Ahn, J.-W. Park, *Materials Chemistry Frontiers* **2024**, *8*, 1861-1887.

[5]	P. E. Stallworth, J. J. Fontanella, M. C. Wintersgill, C. D. Scheidler, J. J. Immel, S. G. Greenbaum, A. S. Gozdz, *J. Power Sources* **1999**, *81-82*, 739-747.

[6]	C. Wang, J. Liang, Y. Zhao, M. Zheng, X. Li, X. Sun, *Energy & Environmental Science* **2021**, *14*, 2577-2619.

[7]	X. Wang, K. He, S. Li, J. Zhang, Y. Lu, *Nano Research* **2022**, *16*, 3741-3765.

[8]	N. Kamaya, K. Homma, Y. Yamakawa, M. Hirayama, R. Kanno, M. Yonemura, T. Kamiyama, Y. Kato, S. Hama, K. Kawamoto, A. Mitsui, *Nat Mater* **2011**, *10*, 682-686.

[9]	P. Vadhva, J. Hu, M. J. Johnson, R. Stocker, M. Braglia, D. J. L. Brett, A. J. E. Rettie, *ChemElectroChem* **2021**, *8*, 1930-1947.

[10]	S. Hori, R. Kanno, X. Sun, S. Song, M. Hirayama, B. Hauck, M. Dippon, S. Dierickx, E. Ivers-Tiffée, *J. Power Sources* **2023**, *556*, 232450.

[11]	M. Petrowsky, R. Frech, *J. Phys. Chem. B* **2009**, *113*, 5996-6000.

[12]	A. Dalvi, N. Parvathala Reddy, S. C. Agarwal, *Solid State Commun.* **2012**, *152*, 612-615.

[13]	B. Darminto, G. J. Rees, J. Cattermull, K. Hashi, M. Diaz-Lopez, N. Kuwata, S. J. Turrell, E. Milan, Y. Chart, C. Di Mino, H. Jeong Lee, A. L. Goodwin, M. Pasta, *Angew. Chem. Int. Ed. Engl.* **2023**, *62*, e202314444.

[14]	J. Kincs, S. W. Martin, *Phys. Rev. Lett.* **1996**, *76*, 70-73.

[15]	J. Kohout, *Molecules* **2021**, *26*, 7162.

[16]	S. Murugavel, *Physical Review B* **2005**, *72*, 134204

[17]	T. Šalkus, E. Kazakevičius, A. Kežionis, A. F. Orliukas, J. C. Badot, O. Bohnke, *Solid State Ionics* **2011**, *188*, 69-72.

[18]	Y. Kato, S. Hori, R. Kanno, *Advanced Energy Materials* **2020**, *10*, 2002153.

[19]	X. Lu, A. Windmuller, D. Schmidt, S. Schoner, C. L. Tsai, H. Kungl, X. Liao, Y. Chen, S. Yu, H. Tempel, R. A. Eichel, *ACS Appl Mater Interfaces* **2023**, *15*, 34973-34982.

[20]	P. Bron, S. Johansson, K. Zick, J. Schmedt auf der Gunne, S. Dehnen, B. Roling, *J. Am. Chem. Soc.* **2013**, *135*, 15694-15697.

[21]	O. Kwon, M. Hirayama, K. Suzuki, Y. Kato, T. Saito, M. Yonemura, T. Kamiyama, R. Kanno, *Journal of Materials Chemistry A* **2015**, *3*, 438-446.

[22]	Y. Okada, M. Ikeda, M. Aniya, *Solid State Ionics* **2015**, *281*, 43-48.

[23]	B. T. Ahn, R. A. Huggins, *Mater. Res. Bull.* **1989**, *24*, 889-897.

[24]	P. Bron, S. Dehnen, B. Roling, *J. Power Sources* **2016**, *329*, 530-535.

[25]	S. P. Culver, A. G. Squires, N. Minafra, C. W. F. Armstrong, T. Krauskopf, F. Bocher, C. Li, B. J. Morgan, W. G. Zeier, *J. Am. Chem. Soc.* **2020**, *142*, 21210-21219.

[26]	C. Dietrich, M. Sadowski, S. Sicolo, D. A. Weber, S. J. Sedlmaier, K. S. Weldert, S. Indris, K. Albe, J. Janek, W. G. Zeier, *Chem. Mater.* **2016**, *28*, 8764-8773.

[27]	S. Hori, K. Suzuki, M. Hirayama, Y. Kato, T. Saito, M. Yonemura, R. Kanno, *Faraday Discuss.* **2014**, *176*, 83-94.

[28]	S. Hori, S. Taminato, K. Suzuki, M. Hirayama, Y. Kato, R. Kanno, *Acta Crystallogr B Struct Sci Cryst Eng Mater* **2015**, *71*, 727-736.

[29]	M. Inagaki, K. Suzuki, S. Hori, K. Yoshino, N. Matsui, M. Yonemura, M. Hirayama, R. Kanno, *Chem. Mater.* **2019**, *31*, 3485-3490.

[30]	Y. Inoue, K. Suzuki, N. Matsui, M. Hirayama, R. Kanno, *J. Solid State Chem.* **2017**, *246*, 334-340.

[31]	T. Kaib, S. Haddadpour, M. Kapitein, P. Bron, C. Schröder, H. Eckert, B. Roling, S. Dehnen, *Chem. Mater.* **2012**, *24*, 2211-2219.

[32]	R. Kanno, T. Hata, Y. Kawamoto, M. Irie, *Solid State Ionics* **2000**, *130*, 97-104.

[33]	R. Kanno, M. Murayama, *J. Electrochem. Soc.* **2001**, *148*, A742.

[34]	Y. Kato, R. Saito, M. Sakano, A. Mitsui, M. Hirayama, R. Kanno, *J. Power Sources* **2014**, *271*, 60-64.

[35]	T. Krauskopf, S. P. Culver, W. G. Zeier, *Chem. Mater.* **2018**, *30*, 1791-1798.

[36]	A. Kuhn, O. Gerbig, C. Zhu, F. Falkenberg, J. Maier, B. V. Lotsch, *Phys. Chem. Chem. Phys.* **2014**, *16*, 14669-14674.

[37]	B. T. Leube, K. K. Inglis, E. J. Carrington, P. M. Sharp, J. F. Shin, A. R. Neale, T. D. Manning, M. J. Pitcher, L. J. Hardwick, M. S. Dyer, F. Blanc, J. B. Claridge, M. J. Rosseinsky, *Chem. Mater.* **2018**, *30*, 7183-7200.

[38]	J. Liang, N. Chen, X. Li, X. Li, K. R. Adair, J. Li, C. Wang, C. Yu, M. Norouzi Banis, L. Zhang, S. Zhao, S. Lu, H. Huang, R. Li, Y. Huang, X. Sun, *Chem. Mater.* **2020**, *32*, 2664-2672.

[39]	H. Lim, S.-C. Kim, J. Kim, Y.-I. Kim, S.-J. Kim, *J. Solid State Chem.* **2018**, *257*, 19-25.

[40]	Z. Liu, F. Huang, J. Yang, B. Wang, J. Sun, *Solid State Ionics* **2008**, *179*, 1714-1716.

[41]	M. Murayama, R. Kanno, M. Irie, S. Ito, T. Hata, N. Sonoyama, Y. Kawamoto, *J. Solid State Chem.* **2002**, *168*, 140-148.

[42]	Y. Sun, K. Suzuki, S. Hori, M. Hirayama, R. Kanno, *Chem. Mater.* **2017**, *29*, 5858-5864.

[43]	M. Tachez, J. Malugani, R. Mercier, G. Robert, *Solid State Ionics* **1984**, *14*, 181-185.

[44]	I. Tarhouchi, V. Viallet, P. Vinatier, M. Ménétrier, *Solid State Ionics* **2016**, *296*, 18-25.

[45]	C. Vinado, S. Wang, Y. He, X. Xiao, Y. Li, C. Wang, J. Yang, *J. Power Sources* **2018**, *396*, 824-830.

[46]	J. M. Whiteley, J. H. Woo, E. Hu, K.-W. Nam, S.-H. Lee, *J. Electrochem. Soc.* **2014**, *161*, A1812-A1817.

[47]	J. Yi, L. Chen, Y. Liu, H. Geng, L. Z. Fan, *ACS Appl Mater Interfaces* **2019**, *11*, 36774-36781.

[48]	J. Yin, X. Yao, G. Peng, J. Yang, Z. Huang, D. Liu, Y. Tao, X. Xu, *Solid State Ionics* **2015**, *274*, 8-11.

[49]	P. Zhou, J. Wang, F. Cheng, F. Li, J. Chen, *Chem Commun (Camb)* **2016**, *52*, 6091-6094.

[50]	Y. Gao, N. Li, Y. Wu, W. Yang, S. H. Bo, *Advanced Energy Materials* **2021**, *11*, 2100325.

[51]	H. Schmidt, M. Wiebe, B. Dittes, M. Grundmann, *Appl. Phys. Lett.* **2007**, *91*, 232110.

[52]	V. W. Meyer, H. Neldel, *Z. Tech. Phys.* **1937**, *12*, 588.

[53]	B. Tao, C. Ren, H. Li, B. Liu, X. Jia, X. Dong, S. Zhang, H. Chang, *Adv. Funct. Mater.* **2022**, *32*, 2203551.

[54]	J. R. Akridge, *U.S. Pat. no. 4465745*, **1984**

[55]	B. Y. Liaw, B. T. Ahn, R. A. Huggins, *Proc. the ECS symposium on Lithium Batteries* **1987**, *87-1*, 393-401.

[56]	J. Malugani, G. Robert, *Solid State Ionics* **1980**, *1*, 519-523.

[57]	D. Ravaine, J. L. Souquet, *J. Chim. Phys.* **1974**, *71*, 693-701.

[58]	C. Ling, *npj Computational Materials* **2022**, *8*, 33.

[59]	J. Janek, W. G. Zeier, *Nature Energy* **2023**, *8*, 230-240.

[60]	S. Hu, C. Huang, *Batteries* **2023**, *9*, 228.

[61]	F. Li, X. Cheng, L. L. Lu, Y. C. Yin, J. D. Luo, G. Lu, Y. F. Meng, H. Mo, T. Tian, J. T. Yang, W. Wen, Z. P. Liu, G. Zhang, C. Shang, H. B. Yao, *Nano Lett.* **2022**, *22*, 2461-2469.

[62]	A. K. Mishra, S. Rajput, M. Karamta, I. Mukhopadhyay, *ACS Omega* **2023**, *8*, 16419-16427.

[63]	B. Zhang, J. Zhong, Y. Zhang, L. Yang, J. Yang, S. Li, L.-W. Wang, F. Pan, Z. Lin, *Nano Energy* **2021**, *79*, 105407.

[64]	H. Zhou, L. Huang, M. Yu, X. Ning, *Energy Storage Materials* **2024**, *70*, 103540.

[65]	K. Fujimura, A. Seko, Y. Koyama, A. Kuwabara, I. Kishida, K. Shitara, C. A. J. Fisher, H. Moriwake, I. Tanaka, *Advanced Energy Materials* **2013**, *3*, 980-985.

[66]	H. Zhou, B. Li, M. Yu, S. Li, G. Fan, X. Ning, *Energy Storage Materials* **2023**, *56*, 205-217.

[67]	L. Breiman, *Machine Learning* **2001**, *45*, 5-32.

[68]	S. Li, J. Lin, M. Schaller, S. Indris, X. Zhang, T. Brezesinski, C. W. Nan, S. Wang, F. Strauss, *Angew. Chem. Int. Ed. Engl.* **2023**, *62*, e202314155.

[69]	J. Lin, G. Cherkashinin, M. Schäfer, G. Melinte, S. Indris, A. Kondrakov, J. Janek, T. Brezesinski, F. Strauss, *ACS Materials Letters* **2022**, *4*, 2187-2194.

[70]	A. Yelon, B. Movaghar, R. S. Crandall, *Rep. Prog. Phys.* **2006**, *69*, 1145-1194.

[71]	A. K. Lavrinenko, T. Famprikis, J. A. Quirk, V. Landgraf, P. B. Groszewicz, J. R. Heringa, S. Smeets, V. Azizi, S. Ciarella, J. A. Dawson, M. Wagemaker, A. Vasileiadis, *J Mater Chem A Mater* **2024**, *12*, 26596-26611.

[72]	M. A. Kraft, S. Ohno, T. Zinkevich, R. Koerver, S. P. Culver, T. Fuchs, A. Senyshyn, S. Indris, B. J. Morgan, W. G. Zeier, *J. Am. Chem. Soc.* **2018**, *140*, 16330-16339.

[73]	F. Zhao, S. Zhang, X. Sun, *Adv. Mater.* **2025**, *37*, e2501544.

[74]	T. Chen, C. Guestrin, Proceedings of the 22nd ACM SIGKDD International Conference on Knowledge Discovery and Data Mining, Association for Computing Machinery, **2016**

[75]	D. E. Rumelhart, G. E. Hinton, R. J. Williams, *Nature* **1986**, *323*, 533-536.

[76]	S. Pereznieto, R. Jaafreh, J.-g. Kim, K. Hamad, *Mater. Lett.* **2023**, *337*, 133926.
